# Integrative approach to sporadic Alzheimer’s disease: deficiency of TYROBP in a tauopathy mouse model reduces C1q and normalizes clinical phenotype while increasing spread and state of phosphorylation of tau

**DOI:** 10.1038/s41380-018-0258-3

**Published:** 2018-10-03

**Authors:** Mickael Audrain, Jean-Vianney Haure-Mirande, Minghui Wang, Soong Ho Kim, Tomas Fanutza, Paramita Chakrabarty, Paul Fraser, Peter H. St George-Hyslop, Todd E. Golde, Robert D. Blitzer, Eric E. Schadt, Bin Zhang, Michelle E. Ehrlich, Sam Gandy

**Affiliations:** 1 Department of Neurology, Icahn School of Medicine at Mount Sinai, New York, NY 10029 USA; 2 Department of Genetics and Genomic Sciences and Icahn Institute of Genomic Sciences, Icahn School of Medicine at Mount Sinai, New York, NY 10029 USA; 3 Department of Neuroscience and McKnight Brain Institute, University of Florida, Gainesville, FL 32610 USA; 4 Tanz Centre for Research in Neurodegenerative Diseases, University of Toronto, Toronto, ON Canada; 5 Departments of Pharmacological Sciences and Psychiatry, Icahn School of Medicine at Mount Sinai, New York, NY 10029 USA; 6 Department of Pediatrics, Icahn School of Medicine at Mount Sinai, New York, NY 10029 USA; 7 Department of Psychiatry and Alzheimer’s Disease Research Center, Icahn School of Medicine at Mount Sinai, New York, NY 10029 USA

**Keywords:** Neuroscience, Molecular biology

## Abstract

TYROBP/DAP12 forms complexes with ectodomains of immune receptors (TREM2, SIRPβ1, CR3) associated with Alzheimer’s disease (AD) and is a network hub and driver in the complement subnetwork identified by multi-scale gene network studies of postmortem human AD brain. Using transgenic or viral approaches, we characterized in mice the effects of TYROBP deficiency on the phenotypic and pathological evolution of tauopathy. Biomarkers usually associated with worsening clinical phenotype (i.e., hyperphosphorylation and increased tauopathy spreading) were unexpectedly increased in *MAPT*^*P301S*^;*Tyrobp*^-/-^ mice despite the improved learning behavior and synaptic function relative to controls with normal levels of TYROBP. Notably, levels of complement cascade initiator C1q were reduced in *MAPT*^*P301S*^;*Tyrobp*^-/-^ mice, consistent with the prediction that C1q reduction exerts a neuroprotective effect. These observations suggest a model wherein TYROBP-KO-(knock-out)-associated reduction in C1q is associated with normalized learning behavior and electrophysiological properties in tauopathy model mice despite a paradoxical evolution of biomarker signatures usually associated with neurological decline.

## Introduction

TYROBP (tyrosine motif binding protein, aka DAP12, DNAX-binding protein-12) has been identified as a causal regulator of multiple genes involved in microglia phagocytosis, and its expression is increased in the brains of patients with Alzheimer’s disease (AD) and mouse models of cerebral amyloidosis [1, 2]. TYROBP is a transmembrane signaling polypeptide and contains an immunoreceptor tyrosine-based activation motif (ITAM) in its cytoplasmic domain. TYROBP serves as an adaptor for, and forms functional complexes with, immune receptor ectodomains, including the triggering receptor expressed on myeloid cells 2 (TREM2) [3], the signal regulatory protein β1 (SIRPβ1) [4] and the complement receptor 3 (CR3) [5, 6]. In the brain, TYROBP is expressed in microglia and dendritic cells. In humans, deletion and nonsense mutations in the *TYROBP* gene cause polycystic lipomembranous osteodysplasia with sclerosing leukoencephalopathy also known as Nasu–Hakola disease [7]. Additionally, missense mutations in *TYROBP* have been reported to be associated with familial AD [8]. Through an integrative network-based approach, members of our group identified *TYROBP* as a key regulator in the complement subnetwork family from late-onset AD (LOAD) [1, 2]. Other investigators have reported that TYROBP is involved in specifying a microglial shift from a homeostatic phenotype to a disease-associated microglia (DAM) phenotype [9]. Along with others, we have reported that absence of TYROBP in amyloid-depositing mouse models reduces microglia clustering around amyloid plaque [10, 11]. Importantly, we showed that constitutive *Tyrobp* deletion in amyloid-depositing mice co-expressing *APP*^*KM670/671NL*^ and *PSEN1*^*Δexon9*^ (*hereafter APP/PSEN1*) prevented learning deficits and electrophysiological abnormalities during the early stages of amyloid deposition [10].

There is a longstanding debate about how much of the neurodegeneration in AD is due to direct proteotoxicity and how much is due to a “neuroinflammation-bystander” effect. Microglia are the main intrinsic cell type implicated in the inflammatory response in the brain and spinal cord. Currently, models hold that microglia can be protective early in the evolution of AD-associated proteinopathy by participating in clearance of protein aggregates, but as the disease progresses, they become inefficient at clearance and release pro-inflammatory cytokines that contribute to neurotoxicity. Although there is an extensive literature on the interaction of microglia with amyloid aggregates and deposits, the interaction of microglia with tauopathy is relatively less extensively studied. Recent studies have shown that depletion of microglia dramatically suppresses propagation of tauopathy and that effect could be exosome dependent since blockage of exosomes also reduced propagation of tauopathy [12, 13]. In addition to the role of microglia in the propagation of tauopathy, lipopolysaccharides-mediated inflammatory stimuli via injection in *MAPT*^*P301S*^ transgenic mice facilitated tau phosphorylation while inducing activation of CD45 and arginase 1 (histological markers of microglia [14]). These results illustrate the role of microglia and inflammation in determining the phosphorylation status of tau.

Because of the reports showing the involvement of microglia in mouse models of tauopathy, we sought to determine the effect of *Tyrobp* deletion on the phenotype of the *MAPT*^*P301S*^ mouse (also known as PS19). In the *Tyrobp*^*-/-*^ mouse, there is a deletion of exons 3 and 4 resulting in loss-of-function of the TYROBP protein by loss of the transmembrane region and part of the cytoplasmic region including the first tyrosine of the ITAM [15]. The *MAPT*^*P301S*^ mice express the human *MAPT* transgene, which encodes the disease-associated P301S mutation and includes four microtubule-binding domains and one N-terminal insert (4R/1N) [16].

The deletion of *Tyrobp* in *MAPT*^*P301S*^ mice led to an unusual constellation of biomarkers and physiological features. Indeed, *MAPT*^*P301*S^;*Tyrobp*^*-/-*^ mice presented an altered phenotype characterized by apparent increases both in the level of tau phosphorylation and in the rapidity of spread of tauopathy through the brain. Despite the conventional wisdom that these biomarker changes would typically be associated with a decline in clinical function, the increased levels of both tau phosphorylation and tauopathy propagation in *MAPT*^*P301*S^;*Tyrobp*^*-/-*^ mice were accompanied by improvement in both clinical and electrophysiological functions. As we reported in our study of *APP/PSEN1*;*Tyrobp*^*-/-*^ mice [10] and our previously reported LOAD network [2], we observed in *MAPT*^*P301*S^;*Tyrobp*^*-/-*^ mice reduced levels of mRNA and protein for C1q, the initiating protein of the classical complement cascade. This consistent association of C1q reduction with *Tyrobp* KO in a variety of crosses suggests a role for TYROBP in the physiological and/or pathological complement-mediated actions of microglia, perhaps including synaptic sculpting [17–20].

## Materials and methods

### Animals

**Fig. 1:**
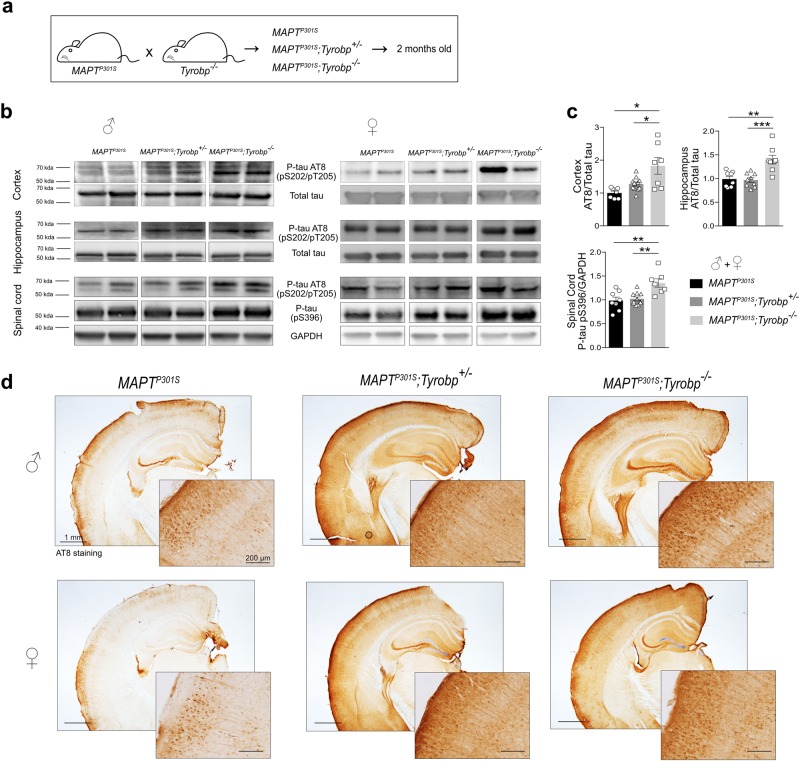
TYROBP deficiency rapidly increases phosphorylated tau levels in a *MAPT*^*P301S*^ transgenic mouse model of tauopathy. **a** *MAPT*^*P301S*^ mice were crossed with *Tyrobp*^*-/-*^ mice, yielding progeny with genotypes of: (1) *MAPT*^*P301S*^ mice expressing wild-type TYROBP (abbreviated *MAPT*^*P301S*^); (2) *MAPT*^*P301S*^ mice heterozygous for *Tyrobp* knock-out (abbreviated *MAPT*^*P301S*^*;Tyrobp*^*+/-*^); (3) *MAPT*^*P301S*^ mice homozygous for *Tyrobp* knock-out (abbreviated *MAPT*^*P301S*^*;Tyrobp*^*-/-*^). Male and female mice (2 months of age) were used for this experiment. **b** Representative western blots of tissues from mice of the indicated genotype were performed using antibodies as indicated: antibody AT8 (detects paired helical filament epitopes of tau phosphorylated at residues serine 202 and/or threonine 205); antibody anti-tau (phospho S^396^) (detects tau phosphorylated at residue serine 396); antibody T46 (detects total tau); anti-GAPDH. Extracts from cortex, hippocampus and spinal cord from male and female mice of the indicated genotypes were studied. Full-length blots are presented in Supplementary Figure 2. **c** Densitometric analyses of western blots standardized to total tau or GAPDH. *n* = 7 (*MAPT*^*P301S*^), *n* = 11 (*MAPT*^*P301S*^*;Tyrobp*^*+/-*^*)* or *n* = 7 (*MAPT*^*P301S*^*;Tyrobp*^*-/-*^) for extracts from cortex; *n* = 8 (*MAPT*^*P301S*^), *n* = 9 (*MAPT*^*P301S*^*;Tyrobp*^*+/-*^*)* or *n* = 8 (*MAPT*^*P301S*^*;Tyrobp*^*-/-*^) for extracts from hippocampus; *n* = 8 (*MAPT*^*P301S*^), *n* = 11 (*MAPT*^*P301S*^*;Tyrobp*^*+/-*^*)* or *n* = 7 (*MAPT*^*P301S*^*;Tyrobp*^*-/-*^) for extracts from spinal cord. Material from male and female mice was pooled for analysis. **d** Representative images of immunohistochemistry with antibody AT8. Hemibrain scale bar = 1 mm; high magnification scale bar = 200 μm. Error bars represent means ± SEM. Statistical analyses were performed using one-way ANOVA followed by Tukey’s post-hoc test, *p < 0.05; **p < 0.01; ***p < 0.001

All experimental procedures were conducted in accordance with the NIH guidelines for animal research and were approved by the Institutional Animal Care and Use Committee (IACUC) at Icahn School of Medicine at Mount Sinai. *MAPT*^*P301S*^ (PS19) and *Tyrobp* knock-out (KO) mice (*Tyrobp*^*-/-*^) were obtained from Jackson Laboratories and Taconic/Merck Laboratory, respectively. *MAPT*^*P301S*^ mice were crossed with *Tyrobp*^*-/-*^ mice to obtain *MAPT*^*P301S*^ mice heterozygous (*MAPT*^*P301S*^*;Tyrobp*^*+/-*^*)* or KO (*MAPT*^*P301S*^*;Tyrobp*^*-/-)*^ for *Tyrobp*. Both males and females were used. TREM2-deficient mice were constructed by targeted homologous recombination [21], which removed exons 1 and 2, which included the start codon and the major extracellular IgG domain. In contrast to the recently reported Velocigene construct, the direction of the Hygromycin cassette was “reversed”. Crucially, in agreement with two other models, but in contrast to the Velocigene construct, reverse transcriptase (RT)-quantitative PCR (qPCR) analyses confirmed (data not shown) specific loss of *Trem2* expression without the perturbation of *Treml1* expression observed in the Velocigene construct [22]. We used a minimum of *n* = 4–5 mice per group (*n* = 7–11 for the Fig. 1, *n* = 6–7 for the behavioral analyses) for most of the analyses as this size gives us 80% power to see differences of effect size of at least 2 between groups at *α* = 0.05. The exact sample size (*n*) for each experiment can be found in each figure legend.

### AAV injection

#### Neonatal mice

WT or *Tyrobp*^*-/-*^ mice at P0 were used in this experiment. Pups were anesthetized on an ice-cold metal plate. Intraventricular brain injection was performed using a Hamilton syringe and 2 μl of virus preparation (AAV8-CBA-MAPT^P301L^, 1.10^12^ vg/site, designated henceforth as AAV-tau) was injected into both hemispheres. Immediately after the injection, pups were placed on a heating blanket. Brains were examined at 6.5 months of age.

#### Adult mice

WT and *Tyrobp*^*-/-*^ mice at 4 months of age were used in this experiment. Mice were anesthetized by an intraperitoneal injection of ketamine/xylazine (80/16 mg/kg body weight) and placed in a stereotactic frame (Stoelting, Wood Dale, IL, USA). Stereotactic intracerebral injections of virus (AAV8-CBA-MAPT^P301L^-GFP, designated AAV-tau-GFP) was performed into layer II/III of medial entorhinal cortex (Ent) bilaterally via the following coordinates: anteroposterior, −4.75 mm; mediolateral, 2.9; dorsoventral, −4.6. We injected 1 μl of virus into each site (1.10^12^ vg/site) at a rate of 0.2 μl/min. Mice were sacrificed at 1, 3, or 6 weeks after injection.

### Tissue collection and sample preparation

Mice were anesthetized in a CO_2_ chamber and then transcardially perfused with 20 ml ice-cold phosphate-buffered saline (PBS). One hemisphere was post-fixed by incubation for 48 h in 4% paraformaldehyde and cut into 35 μm sections with a vibratome (Leica) for histological analyses. The contralateral hemisphere was dissected for isolation of the prefrontal cortex (PFC), hippocampus and cortex. The PFC was used for RNA extraction and RT-qPCR, whereas hippocampal, cortical and spinal cord samples were homogenized in a RIPA buffer (Pierce) containing phosphatase (Pierce) and protease (Roche) inhibitors, centrifuged for 20 min at 15,000 × *g* and the supernatant was used.

### Western blotting

Equal amounts of protein (30 μg) were separated by electrophoresis in precast 4–12% Bis-Tris Gels (Bio-Rad) and transferred to activated/pre-wetted polyvinylidene difluoride membranes. The membranes were hybridized with the following primary antibodies as indicated: AT8 anti-P-tau pSer202/Thr205 (1:500, Thermo Scientific, #MN1020); anti-P-tau pS396 (1:1000, Abcam, #ab109390); anti-total tau T46 (1:1000, Thermo Scientific, #13-6400); anti-GAPDH (1:2000, Santa Cruz, #sc-32233); anti-P-CaMKII (1:1000, Abcam, #ab32678); anti-PSD-95 (1:1000, Millipore, #7E3-1B8), C1q (1:1000, Abcam, #ab182451). Secondary antibodies included: peroxidase-labeled anti-mouse IgG (1:2000, Vector Laboratories); peroxidase-labeled anti-rabbit IgG (1:2000, Vector Laboratories); peroxidase-labeled anti-rat IgG (1:2000, Vector Laboratories). ECL (Pierce®) was used to reveal the immunoreactive proteins, and images were acquired using a Fujifilm ImageReader LAS-4000. Membranes were stripped using a stripping buffer (Thermo Scientific) when required. Luminescent immunoreactive protein bands were quantified using Fiji software (ImageJ).

### Immunohistochemistry and image acquisition

Coronal or sagittal sections (35 µm thickness) were washed with 0.1% Triton in PBS, saturated by incubation with 0.1% Triton in PBS/5% goat serum, and then incubated with primary antibodies as follows: AT8 anti-P-tau pSer202/Thr205 (1/500, Thermo Scientific, #MN1020); anti-P-tau pS396 (1/1000, Abcam, #ab109390); anti-IBA1 (1/1000, Wako, #019-19741), CD68 (1/500, Bio-Rad, #MCA1957) and anti-C1q (1/1000, Abcam, #ab182451). For fluorescent immunostaining, sections were incubated with the appropriate secondary antibody: anti-rabbit IgG Alexa Fluor 488 or 568 (1/2000, Invitrogen); anti-mouse IgG Alexa Fluor 488 or 568 (1/2000, Invitrogen). For non-fluorescent immunostaining, endogenous peroxidase was quenched with PBS containing 3% H_2_O_2_ for 15 min followed by amplification using the ABC system (VECTASTAIN Elite ABC HRP Kit, Vector Laboratories, Burlingame, CA, USA). Horseradish peroxidase conjugates and 3,3′-diaminobenzidine were used according to the manufacturer’s manual (Vector® DAB, Vector Laboratories, Burlingame, CA, USA). Images were obtained with an Olympus BX61 microscope and analyzed with Fiji software (ImageJ).

### Behavioral assessment

#### Barnes maze

Six- and 10-month-old mice were transported from their cage to the center of the platform via a closed starting chamber where they remained for 10 s prior to exploring the maze for 3 min. Mice failing to enter the escape box within 3 min were guided to the escape box by the experimenter, and the latency recorded was noted as 180 s. Mice were allowed to remain in the escape box for 1 min before the next trial. Two trials per day during 4 consecutive days were performed. The platform and the escape box were wiped with 70% ethanol after each trial to eliminate any residual olfactory cues that could enable location of the target hole. All trials were recorded by video camera and analyzed with ANY-maze video tracking software (Stoelting Co., Wood Dale, USA).

#### Novel object recognition

The tests were performed in a white rectangular open field (36 × 26 × 22.5 cm) with sawdust bedding. During the training phase, mice were placed in the experimental apparatus with two different objects (100 mL beakers; blue Lego blocks) and were allowed to explore for 10 min. Objects were cleaned with ethanol between trials to remove olfactory cues. Long-term learning behavior (retention test) was tested 24 h after training on the following day. During the retention test, mice explored the experimental apparatus for 5 min with one familiar and one novel object. All trials were recorded by video camera and analyzed with ANY-maze video tracking software (Stoelting Co., Wood Dale, USA). The behavior assessment was performed with the operator blinded to mouse identity.

### Ex vivo electrophysiology

Coronal brain slices containing the hippocampal formation were prepared as previously described [23]. Animals were anesthetized with isoflurane and brains were rapidly removed from the skull and placed in an ice-cold modified artificial cerebrospinal fluid (ACSF) solution containing: 215 mM sucrose, 2.5 mM KCl, 1.6 mM NaH_2_PO_4_, 4 mM MgSO_4_, 1 mM CaCl_2_, 4 mM MgCl_2_, 20 mM glucose, 26 mM NaHCO_3_ (pH = 7.4, equilibrated with 95% O_2_ and 5% CO_2_). Coronal brain slices (400 µm thick) were prepared with a Vibratome VT1000S (Leica Microsystems, Germany) and then incubated at room temperature for ≥ 3 h in a physiologic ACSF, containing: 120 mM NaCl, 3.3 mM KCl, 1.2 mM Na_2_HPO_4_, 26 mM NaHCO_3_, 1.3 mM MgSO_4_, 1.8 mM CaCl_2_, 11 mM glucose (pH = 7.4 equilibrated with 95% O_2_ and 5% CO_2_). The hemi-slices were transferred to a recording chamber perfused with ACSF at a flow rate of ~2 mL/min using a peristaltic pump; experiments were performed at 28.0 ± 0.1 °C. Recordings were acquired with a GeneClamp 500B amplifier and Digidata 1440A (Molecular Devices). All signals were low-pass filtered at 2 kHz and digitized at 10 kHz. For extracellular field recordings (field excitatory postsynaptic potential (fEPSP) recordings), a patch-type pipette was fabricated on a micropipette puller (Sutter Instruments), filled with ACSF, and placed in the middle third of stratum radiatum in area CA1. fEPSPs were evoked by activating Shaffer collaterals with a concentric bipolar electrode (FHC, Inc.) placed in the middle third of stratum radiatum 150–200 µm away from the recording pipette. Square-wave current pulses (60 µs pulse width) were delivered through a stimulus isolator (Isoflex, AMPI). Input–output curves were generated by a series of stimuli in 0.1 mA steps. Long-term potentiation (LTP) was induced after 20 min of stable baseline recordings (one stimulus every 30 s), using stimuli that yielded a response equal to 30–40% of spike threshold, by theta burst stimulation, which consisted in a series of 10 bursts of 4 stimuli (100 Hz within the burst, 200 ms interburst interval) repeated four times (10 s apart) and delivered at an intensity that yielded a baseline response equal to 75% of spike threshold. Long-term depression (LTD) was induced after 20 min of stable baseline recording by treating the slices with (RS)-3, 5-dihydroxyphenylglycine (DHPG), a selective mGluR 1/mGluR 5 agonist, at 100 µM for 5 min. fEPSPs were collected for 60 min after the start of DHPG. All recordings were performed blind.

### Molecular biological analyses

#### RT-qPCR

RNAs were isolated from PFCs using the QIAzol® Lysis Reagent (Qiagen) and the miRNeasy® Mini Kit (Qiagen). For RT-qPCR analyses, the abundance of each transcript of interest was normalized to the abundance of L32 with the ΔCt method. The All-in-One qPCR Mix (GeneCopoeia) was used to perform RT-qPCR. The sequences of oligonucleotides used:

C1q: Fwd 5′-AACCTCGGATACCAGTCCG-3′; Rev 5′-ATGGGGCTCCAGGAAATC-3′;

TREM2: Fwd 5′-GAGTCAATCCAGCCTGCATG-3′; Rev 5′-TGACAGACAACCATCCAGCT-3′;

CD68: Fwd 5′-TGTCTGATCTTGCTAGGACCG-3′; Rev 5′-GAGAGTAACGGCCTTTTTGTGA-3′.

#### RNA sequencing

RNA-seq assays were performed using total RNAs after ribosomal RNA depletion by Ribo-Zero. In all, 100-nt single-end reads were generated via the Illumina HiSeq 2500 system. Sequencing reads were aligned to mouse reference genome mm10 (GRCm38.90) using STAR aligner guided by mouse GENCODE gene model release v15. All processed total RNA samples had RQN/RIN value of 8.2 or greater. To confirm the expression of human tau gene in the *MAPT*^*P301S*^ mice, a pseudochromosome with the human *MAPT* gene sequence was inserted into the mouse reference genome. Accepted mapped reads were summarized to gene levels using the featureCounts program. Raw count data were normalized by the voom function in the R limma package and then differential expression was identified by the moderated *t*-test implemented in limma. Differentially expressed genes (DEGs) were defined to have at least 1.2-fold change in expression and Benjamini–Hochberg [24] adjusted *p* ≤ 0.1 comparing different genotypes.

### Statistics

Graphs represent the mean of all samples in each group ± SEM. Sample sizes (*n* values) and statistical tests are indicated in figure legends. A Shapiro–Wilk normality test with *α* = 0.05 was used. Analyses used include one-way analysis of variance (ANOVA), two-way ANOVA, or Student's *t*-test. For ANOVA analyses, Tukey's post-hoc tests were used. Significance is reported at *p* ≤ 0.05. Grubb’s test for outliers was used with *α* = 0.05.

## Results

### Microglial TYROBP deficiency is associated with increased hyperphosphorylation of tau in transgenic and virus-induced mouse models of tauopathy

**Fig. 2:**
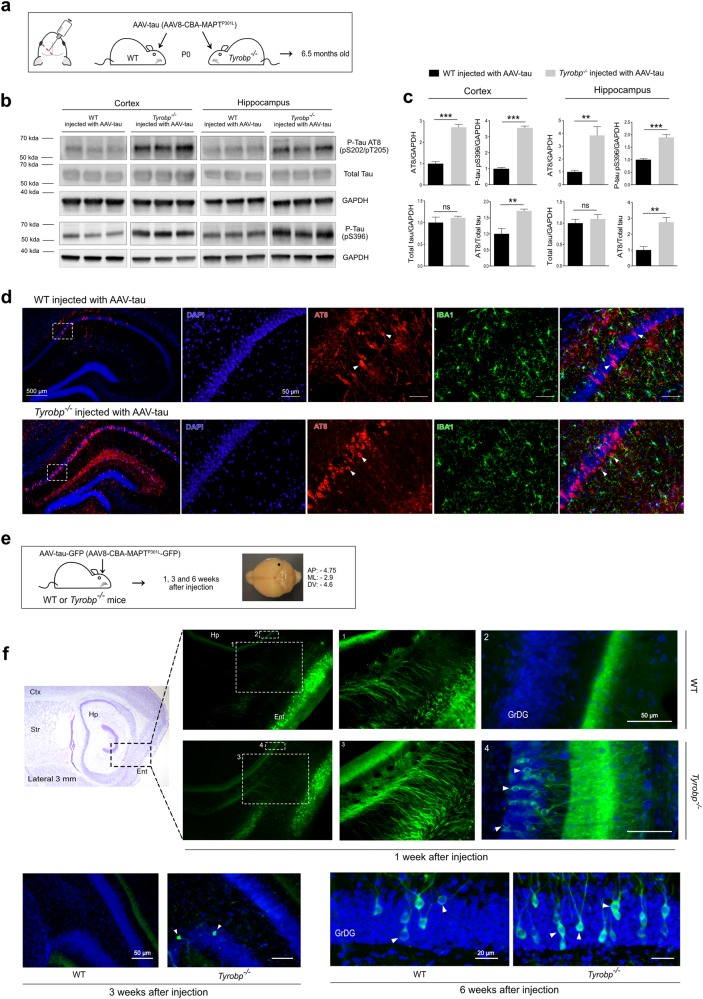
TYROBP deficiency promotes tau phosphorylation and spread in AAV-tau-based mouse models of tauopathy. **a** Wild-type or *Tyrobp*^*-/-*^ P0 pups received intracerebroventricular injections of AAV8-Tau^P301L^ (designated AAV-tau) using a chicken β-actin (CBA) promoter. Brains were examined at age 6.5 months. **b** Representative western blots of extracts from cortical and hippocampal regions of wild-type (WT) or *Tyrobp*^*-/-*^ mice injected with AAV-tau using antibodies as indicated: antibody AT8 (detects paired helical filament epitopes of tau phosphorylated at residues serine 202 and/or threonine 205); antibody anti-tau (phospho S^396^) (detects tau phosphorylated at residue serine 396); antibody T46 (detects total tau); anti-GAPDH. Full-length blots are presented in Supplementary Figure 2. **c** Densitometric analyses of P-tau western blots standardized to GAPDH or total tau. *n* = 5 (*WT* and *Tyrobp*^*-/-*^*)* for extracts from hippocampus and *n* = 4 (*WT*) or *n* = 5 (*Tyrobp*^*-/-*^*)* for extracts from cortex. Error bars represent means ± SEM. Both males and females were used, and analysis was performed on combined results. Statistical analyses were performed using a Student’s *t*-test, **p < 0.01; ***p < 0.001; ns nonsignificant. **d** High magnification of coronal sections of hippocampi showing increased AT8 staining in *Tyrobp*^*-/-*^ mice injected with AAV-tau. Scale bar: hippocampus = 500 μm and high magnification = 5 μm. **e** AAV8-Tau^P301L^-GFP (designated AAV-tau-GFP) was injected in the medial entorhinal cortex to study the spread of tau from this structure to the hippocampus. AP anteroposterior, ML mediolateral, DV dorsoventral. The perforant pathway connecting these two regions is implicated in this spread, although the diffuseness of the immunoreactivity raises the possibility that spread may occur via the interstitium in addition to (or instead of) via neuronal uptake and transneuronal propagation along the classical neuroanatomic pathway. **f** Wild-type or *Tyrobp*^*-/-*^ mice were injected with AAV-tau-GFP at 4 months of age, and brains were examined at 1, 3 or 6 weeks after injection (*n* = 3 per group). Males and females were used for experiments, and results were combined for analysis. Ctx cortex, Str striatum, Hp hippocampus, Ent entorhinal cortex, GrDG granule cell layer of the dentate gyrus

We first assessed the connection between microgliosis and tau pathology using anti-IBA1 (for microglia) and AT8 (for hyperphosphorylated tau on epitopes pS202/pT205) on WT and *MAPT*^*P301S*^ mice, which express a human microtubule-associated tau (MAPT) harboring the P301S mutation linked to familial tauopathy [16]. Although microglia displayed activated morphology in *MAPT*^*P301S*^ mice compared with WT (Supplementary Figure 1a), we mostly observed an intense microgliosis in brain areas exhibiting neurons with dense hyperphosphorylated tau (Supplementary Figure 1b), thereby confirming that major microglial involvement can accompany tauopathy. We evaluated tau phosphorylation status in the hippocampus, cortex and spinal cord of 2-month-old male and female *MAPT*^*P301S*^ mice that are either wild-type (WT), heterozygous or homozygous KO for *Tyrobp* (Fig. 1a). Western blot analyses using AT8 and pS396 antibodies (Fig. 1b) revealed increased levels of phospho-tau in all three regions and in both males and females KO for TYROBP (Fig. 1c), whereas total tau levels were unchanged. Increased phospho-tau within brains from *MAPT*^*P301S*^;*Tyrobp*^*+/-*^ and *MAPT*^*P301S*^;*Tyrobp*^*-/-*^ mice was further confirmed immunohistochemically (Fig. 1d). Because similar results were observed in males and females, both sexes were included in the follow-up experiments. To confirm these results using another modeling strategy and another *MAPT* mutation, we used intracerebroventricular injections of an adeno-associated virus (AAV) with the chicken β-actin (CBA) promoter directing expression of the human *MAPT* complementary DNA harboring the P301L mutation (designated AAV-tau). We injected WT and *Tyrobp*^*-/-*^ P0 pups and characterized them at 6.5 months (Fig. 2a). Quantification of pS202/pT205 and pS396 phospho-epitopes within both hippocampus and cortex demonstrated increased levels of phospho-tau in *Tyrobp*^*-/-*^ mice injected with AAV-tau as compared with the levels in the WT mice injected with AAV-tau (Fig. 2b, c). Consistent with this result, we also observed an accumulation of phosphorylated-tau in CA1 hippocampal neurons by immunohistochemistry in these same mice (Fig. 2d). Thus, two different modeling strategies demonstrated that TYROBP deficiency leads to elevated levels of phospho-tau. Having established that TYROBP deficiency or absence increases tau phosphorylation, we evaluated the potential involvement of selected protein kinases such as GSK3β, CDK5 or DYRK1A. No differences between levels of the transcripts for these enzymes were observed by immunoblotting or RT-qPCR when *MAPT*^*P301S*^ mice were compared against *MAPT*^*P301S*^*;Tyrobp*^*-/-*^ mice (data not shown).

It is well known that microglia form a physical barrier around amyloid plaques, and that barrier is disrupted in the absence of *Tyrobp* [11]. Thereby, we predicted that a deletion of *Tyrobp* in *MAPT*^*P301S*^ mice could alter the spread of soluble forms of tau. To test this hypothesis, we injected AAV encoding *MAPT*^*P301L*^ gene tagged with the green fluorescent protein (GFP) (AAV-tau-GFP) in the entorhinal cortex of 4-month-old mice WT or *Tyrobp*^-/-[12]^ (Fig. 2e). GFP expression was identified in neurons in the entorhinal cortex and perforant pathway in the first week following injection (Supplementary Figure 3). In *Tyrobp*^*-/-*^ mice injected with the AAV-tau-GFP, several GFP-positive neurons were evident in the granular layer of the dentate gyrus (GrDG) beginning 1 week after injection, but no cells were detectable at this stage in the injected WT mice (Fig. 2f). Consistent with this observation, we observed GFP-positive neurons in a more medial GrDG in the *Tyrobp*^*-/-*^ mice, 3 weeks after injection. We also observed GFP-positive neurons in the GrDG of *Tyrobp*^*-/-*^ mice 6 weeks after injection and, to a lesser extent, in WT mice (Fig. 2f). These data indicate that an absence of TYROBP promotes the spread of tau. This tau spreading phenomenon probably accounts for the diffuse pattern of increased AT8 staining (see Figs. 1d, 2d).

### TYROBP deficiency is associated with an altered microglial phenotype

**Fig. 3:**
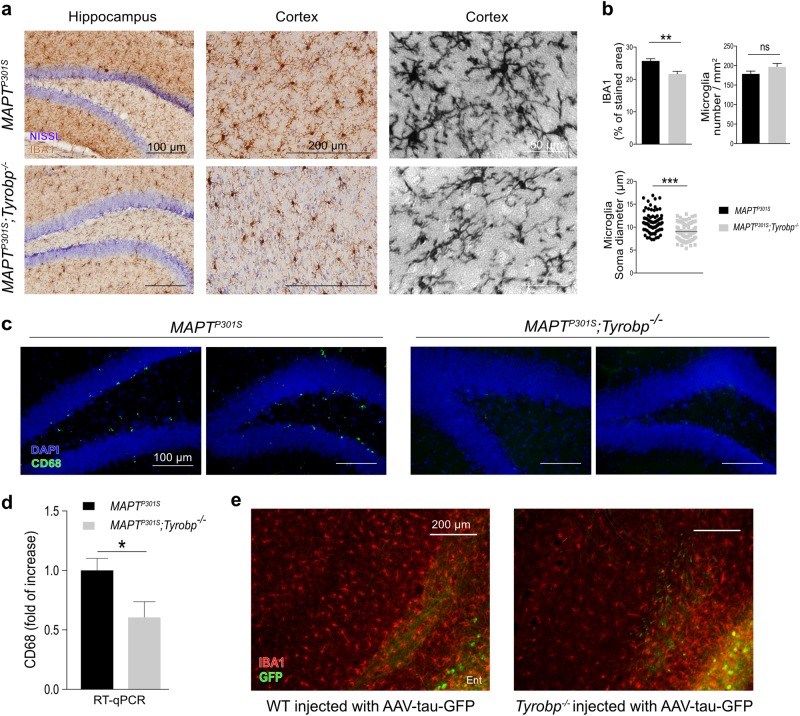
TYROBP deficiency alters microglia phenotype. **a** Representative images of anti-Iba1 DAB immunohistochemistry in 4-month-old *MAPT*^*P301S*^ mice with (top) and without (bottom) TYROBP. Scale bars = 100, 200 and 50 μm, respectively. **b** Top left panel: quantification of IBA1 immunoreactivity in cortical areas (*n* = 4 mice per group with an average of 4–5 slices per mouse). Top right panel: average microglia density (cells/mm^2^; *n* = 4 mice per group with an average of 4–5 slices per mouse). Bottom panel: diameter of microglial soma in cortex. *n* = 20 microglia per mouse and area with *n* = 4 (*MAPT*^*P301S*^) or *n* = 5 (*MAPT*^*P301S*^*;Tyrobp*^*-/-*^) mice per group. **c** Representative images of anti-CD68 immunohistochemistry in the hippocampus of the same groups as in Fig. 3a. Scale bar = 100 μm. **d** RT-qPCR of CD68 mRNA in the prefrontal cortex in the same mice as in Fig. 3a with *n* = 4 mice per group. **e** Representative images of immunohistochemistry with anti-Iba1 antibody in WT or *Tyrobp-*null mice injected with AAV-tau-GFP in the medial entorhinal cortex (see Fig. 2 and Supplementary Figure 3). Scale bar = 200 μm. Error bars represent means ± SEM. Males and females were used for experiments, and results were combined for analysis. Statistical analyses were performed using a Student’s *t*-test, *p < 0.05; **p < 0.01; ***p < 0.001

We previously showed by immunohistochemistry and flow cytometry that a deficiency of TYROBP in a WT background does not modify microglia number and phenotype in 4-month-old WT mice [10]. To determine whether the increased level and diffusion of phosphorylated tau in *MAPT*^*P301S*^ mice KO for *Tyrobp* is associated with an altered microglial morphology, we performed anti-IBA1 immunohistochemistry on 4-month-old *MAPT*^*P301S*^ mice KO or WT for *Tyrobp* (Fig. 3a). There was a marked reduction in IBA1 staining intensity, as well as a reduction of the soma diameter in *MAPT*^*P301S*^*;Tyrobp*^*-/-*^ microglia as compared with *MAPT*^*P301S*^ with normal level of TYROBP (Fig. 3b). Moreover, we observed in the hippocampus of *MAPT*^*P301S*^*;Tyrobp*^*-/-*^ a dramatic reduction in the immunocytochemical signal for CD68, a lysosomal-localized indicator of microglial phagocytic activity (Fig. 3c) [25, 26]. We confirmed the reduction of CD68 protein at the mRNA level by RT-qPCR in the PFC of *MAPT*^*P301S*^ *and MAPT*^*P301*^*;Tyrobp*^*-/-*^ (Fig. 3d). We also noticed a decrease of the IBA1 intensity in the *Tyrobp*^-/-^ mice injected with the AAV-tau-GFP in the entorhinal cortex (Fig. 3e). This was similar to the effect that we observe when AAV-tau was injected at P0 (see Fig. 2d).

### Deletion of *Tyrobp* in *MAPT*^*P301S*^ mice is associated with reduction in levels of key complement protein C1q

**Fig. 4:**
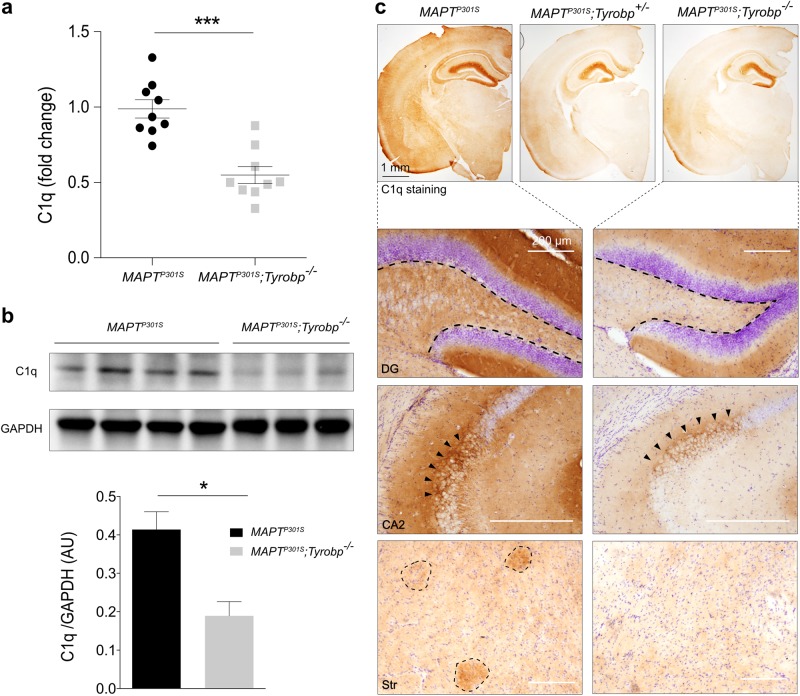
TYROBP deficiency is associated with decreased C1q RNA and protein levels. **a** RT-qPCR analysis for C1q in *MAPT*^*P301S*^ mice with normal or absent TYROBP. Assay was performed on prefrontal cortex samples from males and females. *n* = 9 mice per group. **b** Western blot and densitometric analysis of C1q protein in cortical samples. *n* = 4 (*MAPT*^*P301S*^) or *n* = 3 (*MAPT*^*P301S*^*;Tyrobp*^*-/-*^) mice per group. **c** Representative images of anti-C1q immunohistochemistry, showing intense immunoreactivity in cells of the hippocampus and in a nerve terminal-like pattern in the dentate gyrus (DG) and in region CA2. C1q-immunopositive patches are visualized in the cortex and striatum. Scale bar = 200 μm. Immunostaining intensity in all regions is decreased in the indicated regions of *Tyrobp*^-/-^ mice. Males and females (4 months of age) were used for experiments, and results were combined for analysis. Error bars represent means ± SEM. Statistical analyses were performed using a Student’s *t*-test, *p < 0.05; ***p < 0.001

*TYROBP* has been identified in LOAD patients as a key regulator of the complement subnetwork [2]. C1q is the recognition component of C1, the classical complement pathway multi-subunit complex, and is a member of the complement subnetwork regulated by *TYROBP* in AD patients [2]. Microglia are the main source of C1q in the mouse brain [27]. We performed RT-qPCR in the PFC of 4-month-old *MAPT*^*P301S*^ mice WT or KO for *Tyrobp*. Absence of TYROBP was associated with a significant reduction in the levels of *C1q* mRNA (Fig. 4a). We confirmed the decreased C1q expression by western blot (Fig. 4b). Notably, the areas of reduced C1q-like immunoreactivity in the brain of *MAPT*^*P301S*^;*Tyrobp*^*-/-*^ mice were regions that are particularly rich in synapses, including hippocampal CA2 and dentate gyrus [28]. We also observed an apparent reduction in the extent to which C1q-positive patches were detected in the cortex and striatum (Fig. 4c) [29]. These results indicate that TYROBP deficiency leads to a reduction in levels of the complement system protein C1q in tauopathy mice. This effect is the opposite of situation in brain aging and AD where C1q levels are abnormally elevated [28, 29].

### TREM2 deficiency exacerbates tauopathy without causing a reduction in levels of C1q RNA or protein

TYROBP is the direct adaptor of TREM2 and variants in the *TREM2* gene increase the risk for developing AD [30, 31]. However, the role of TREM2 in AD and tauopathy are not yet fully understood. One recent report showed that TREM2 deficiency could attenuate tauopathy, thereby resulting in apparent benefits as measured by functional or biomarker assays [32], whereas another showed that TREM2 deficiency exacerbated tauopathy [33]. We compared *MAPT*^*P301S*^ mice on a WT background with those on a *Trem2*-null or *Tyrobp*-null background at 4 months of age. As above, the stoichiometry of phospho-tau standardized to total tau was elevated in *MAPT*^*P301S*^*;Tyrobp*^-/-^ extract but unchanged in *MAPTP*^*301S*^*;Trem2*^*-/-*^ mice (Supplementary Figure 4a, b). We confirmed these results by immunohistochemistry using a second antibody, which recognizes the phosphorylated pS396 epitope of tau (Supplementary Figure 4c). Interestingly, at 8 months of age, we were able to detect an increased level of phospho-tau in *MAPTP*^*301S*^*;Trem2*^*-/-*^ mice as compared with *MAPT*^*P301S*^ mice (Supplementary Figure 4d). However, we did not observe any reduction of C1q protein or mRNA in *MAPTP*^*301S*^*;Trem2*^*-/-*^ as compared with *MAPTP*^*301S*^ (Supplementary Figure 4e, f). Together, these data indicate that increased tau phosphorylation results from either TYROBP or TREM2 deficiency, but the two follow different time courses, perhaps explaining the apparent contradictions in the literature among some recent studies. Deficiency of TREM2, however, did not lead to a reduction in C1q levels.

### Deletion of *Tyrobp* is associated with improved behavioral performance

**Fig. 5:**
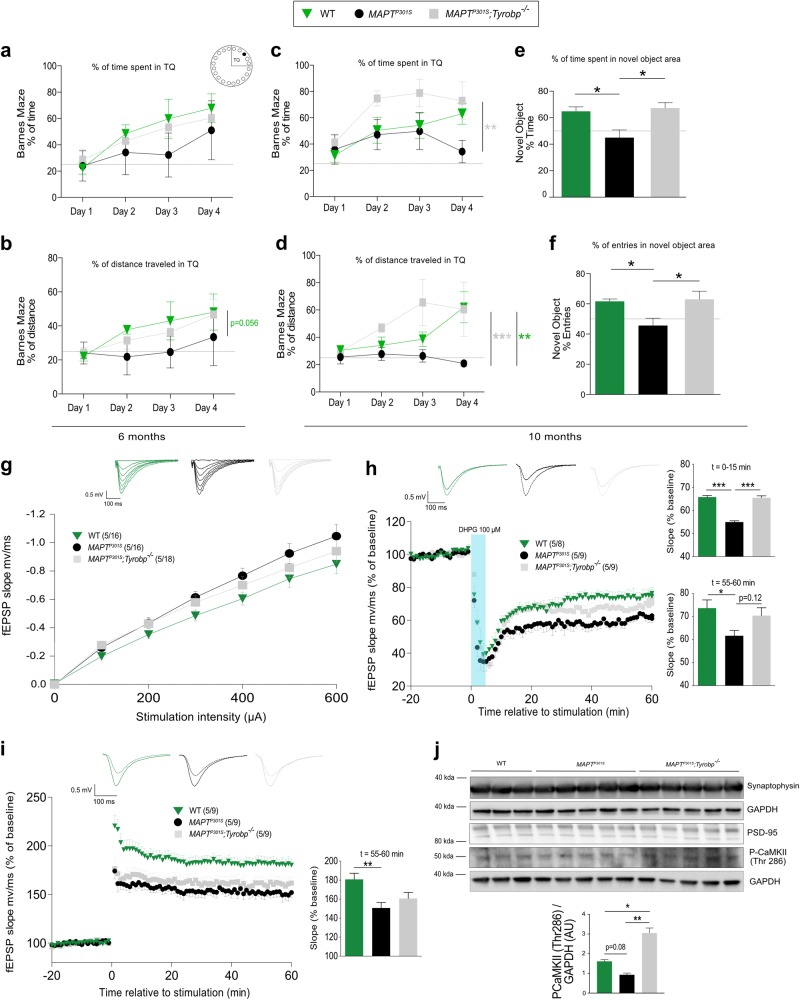
TYROBP deficiency enhances Barnes Maze and Novel Object Recognition performances and reduces synaptic impairments. **a**, **b** Wild-type and *MAPT*^*P301S*^ mice with and without knock-out of *Tyrobp* were tested at 6 months of age in the Barnes Maze. Training was performed on 4 days with two trials per day, and the percentage of time (**a**) or distance (**b**) spent in the target quadrant (TQ) containing the entry for the escape zone is located were measured. **c**, **d** Barnes Maze testing was performed in the same cohort at 10 months of age. **e**, **f** The same mice used in Figs. 5a–d were tested with the Novel Object Recognition task at 10 months of age. The percentage of time spent in the novel object area (**e**), as well as the percentage of entries into the novel object area (**f**) were measured. The dotted lines represent 25 and 50% for Figs. 5 a–d, e–f, respectively. *n* = 6 (*WT*), *n* = 7 (*MAPT*^*P301S*^*)* or *n* = 7 (*MAPT*^*P301S*^*;Tyrobp*^*-/-*^) mice per group. Error bars represent the means ± SEM. A two-way ANOVA corrected for multiple comparisons by a Tukey’s post-hoc test was used for Figs. 5a–d. A one-way ANOVA followed by a Tukey’s post-hoc test was used for Figs. 5e, f, *p < 0.05; **p < 0.01; ***p < 0.001. Males and females were used for experiments, and results were combined for analysis. **g** Input–output relationship measuring basal synaptic function in 11-month-old wild-type (WT; *n* = 5 mice; 16 recordings), *MAPT*^*P301S*^ (*n* = 5; 16 recordings) and *MAPT*^*P301S*^*;Tyrobp*^*-/-*^ (*n* = 5 mice; 18 recordings) mice. **h** Long-term depression (LTD) was induced using (RS)-3,5-dihydroxyphenylglyine (DHPG) in mice of the same genotype series described above: WT (*n* = 5 mice; 8 recordings), *MAPT*^*P301S*^ (*n* = 5 mice; 9 recordings) and *MAPT*^*P301S*^*;Tyrobp*^*-/-*^ (*n* = 5 mice; 9 recordings) mice. Mean values for the first 15’ after DHPG induction or for the last 5’ after DHPG induction are presented in the panels on the right side of the figure. **i** Synaptically induced long-term potentiation (LTP) over 60 min was induced by high-frequency stimulation in mice of the same genotype series described above: WT (*n* = 5 mice; 9 recordings), *MAPT*^*P301S*^ (*n* = 5; 9 recordings) and *MAPT*^*P301S*^*;Tyrobp*^*-/-*^ (*n* = 5 mice; 9 recordings) mice. For panels **g**-**i**, representative traces are shown on the top. **j** Western blots for synaptophysin, calcium/calmodulin-dependent protein kinase II (CaMKII) phosphorylated on threonine 286, PSD-95 and GAPDH were performed on extracts of prefrontal cortex from the same cohorts: WT (*n* = 3), *MAPT*^*P301S*^ (*n* = 5) and *MAPT*^*P301S*^*;Tyrobp*^*-/-*^ (*n* = 5). Extracts were prepared from samples of prefrontal cortex removed prior to slicing for the electrophysiological recordings. Error bars represent means ± SEM. Males and females were used for experiments, and results were combined for analysis. Statistical analyses were performed using a one-way ANOVA followed by a Tukey’s post-hoc test, *p < 0.05; **p < 0.01; ***p < 0.001

We recently showed that the functional status of a mouse model of amyloidosis is sustained in the presence of a genetic *Tyrobp* deletion [10]. Moreover, it has been suggested that the microglial- and complement-dependent pathways that prune excess synapses in development are inappropriately activated and mediate synapse loss in AD [20, 28, 29]. Therefore, we hypothesized that the decrease of C1q observed in *MAPT*^*P301S*^ mice deficient for TYROBP may also correlate with beneficial effects on learning and memory. We first evaluated spatial learning and memory using the Barnes Maze task on 6-month-old WT, *MAPT*^*P301S*^ and *MAPT*^*P301S*^;*Tyrobp*^*-/-*^ mice. Both percentages of time spent and distance traveled in the target quadrant (TQ), the location of the hole for the escape box, were higher in WT and *MAPT*^*P301S*^;*Tyrobp*^*-/-*^ mice compared with *MAPT*^*P301S*^ mice (Fig. 5a, b). Despite that difference, *MAPT*^*P301S*^ mice spent >25% of time and distance in TQ meaning that the deficit in these mice at this age is present, albeit subtle. Because approximately 80% of *MAPT*^*P301S*^ die by 12–13 months of age [16], we also assayed 10-month-old mice in the same task. Although the impairment is worsened in *MAPT*^*P301S*^ at this age, *MAPT*^*P301S*^;*Tyrobp*^*-/-*^ mice spent >60% of time and distance in TQ on the last day of training (Fig. 5c, d). We also tested the mice on the Novel Object Recognition task often used to evaluate working memory, attention, anxiety and preference for novelty in rodents [34, 35]. *MAPT*^*P301S*^*;Tyrobp*^*-/-*^ mice spent significantly more time in the novel object area when compared with the time spent in the area of the familiar object, relative to *MAPT*^*P301S*^ mice (Fig. 5e, f). Consistent with our previous report in an amyloidosis mouse model of AD [10], *Tyrobp* deletion prevents the development of functional and behavioral changes in *MAPT*^*P301S*^ mice.

### TYROBP deficiency alters synaptic pathophysiology in *MAPT*^*P301S*^ mice

We next investigated synaptic plasticity in the absence of *Tyrobp* in *MAPT*^*P301S*^ mice. Basal synaptic efficiency was measured by determining the slope of input–output relationship in WT, *MAPT*^*P301S*^ and *MAPT*^*P301S*^;*Tyrobp*^*-/-*^ mice at 11 months. Absence of TYROBP in *MAPT*^*P301S*^ partially restored the abnormal basal synaptic activity observed in *MAPT*^*P301S*^ mice (Fig. 5g). We used a synaptic induction protocol to induce a prominent protein synthesis-dependent “late” phase of LTD. Slices from *MAPT*^*P301S*^ mice showed significantly impaired LTD compared with WT, and absence of TYROBP in *MAPT*^*P301S*^ mice restored the initial phase of the LTD (Fig. 5h). LTP was also altered in *MAPT*^*P301S*^ slices as compared with WT, but there was only a trend for restoration in the absence of TYROBP (Fig. 5i). Although synaptic plasticity as measured by LTP was not fully restored, the level of calcium/calmodulin-dependent protein kinase II (CaMKII), a protein kinase that potentiates synaptic transmission and whose autophosphorylation on threonine 286 is crucial for LTP induction [36], was increased in *MAPT*^*P301S*^;*Tyrobp*^*-/-*^ mice relative to *MAPT*^*P301S*^ (Fig. 5j). In summary, these data indicate that *Tyrobp* deletion in a *MAPT*^*P301S*^ tauopathy model improves synaptic plasticity, similar to what we observed in a mouse model of cerebral amyloidosis [10].

### Gene differential expression in WT, *MAPT*^*P301S*^ and *MAPT*^*P301S*^;*Tyrobp*^*-/-*^ mice at 11 months of age

In order to identify DEGs in *MAPT*^*P301S*^;*Tyrobp*^*-/-*^ mice and to compare them with the network previously obtained by bulk RNA-seq from sporadic LOAD patients [2], we generated transcriptomic profiles from the PFC of 11 months old females WT, *MAPT*^*P301S*^ and *MAPT*^*P301S*^;*Tyrobp*^*-/-*^ mice (*N* = 4 of each genotype). RNA sequence reads mapped to the human *MAPT* gene confirmed its overexpression in *MAPT*^*P301S*^ mice deficient or WT for TYROBP (Supplementary Figure 5a). We identified 69 DEGs in *MAPT*^*P301S*^;*Tyrobp*^*-/-*^ mice compared with *MAPT*^*P301S*^ mice. In comparison with WT mice, 14 DEGs (all upregulated) were identified in *MAPT*^*P301S*^ mice, whereas 127 DEGs were identified in *MAPT*^*P301S*^;*Tyrobp*^*-/-*^ mice (Supplementary Figure 5b, c; FDR ≤ 0.1; FC ≥ 1.2). All the 14 upregulated DEGs in *MAPT*^*P301S*^ mice were also significantly upregulated in *MAPT*^*P301S*^;*Tyrobp*^*-/-*^ mice (Supplementary Figure 5g). Overall, we observed 23 downregulated and 22 upregulated DEGs common between the *MAPT*^*P301S*^;*Tyrobp*^*-/-*^ vs *MAPT*^*P301S*^ and the *MAPT*^*P301S*^;*Tyrobp*^*-/-*^ vs WT comparisons (Supplementary Figure 5d–g). Apart from *Tyrobp* that is the top downregulated DEG, *Bub1b* and *Nudt19* that have been proposed as early biomarkers of AD [37] were in the top 10 DEGs (Supplementary Figure 5d, e). Only 14 upregulated DEGs were identified when comparing *MAPT*^*P301S*^ and WT mice, and we previously identified only 11 and 21 DEGs at 4 and 8 months of age, respectively, in PFC samples from WT and *Tyrobp*^*-/-*^ mice (data not shown). Together, these data indicate that absence of TYROBP does not modify the transcriptome in the absence of another stressor at the large effect sizes we were powered to see with our small sample sizes. Interestingly, the 24 DEGs between *MAPT*^*P301S*^;*Tyrobp*^*-/-*^ and *MAPT*^*P301S*^ (on 69) did not overlap with those between *MAPT*^*P301S*^;*Tyrobp*^*-/-*^ and WT comparison (Supplementary Table 1), suggesting they were specifically associated with *Tyrobp* deletion in the context of a tauopathy. *Snrpf* (logFC = −0.5) is a core component of U1, U2, U4 and U5 small nuclear ribonucleoproteins and is downregulated in *MAPT*^*P301S*^;*Tyrobp*^*-/-*^ mice. Decreased SNRPF levels have been recently associated with the AD-risk variant rs4420638 [38] and knockdown of SNRPF leads to aggregation of huntingtin protein [39]. Haus5 (logFC = −0.5) is a subunit of the augmin (or Haus) complex, a microtubule-binding complex involved in microtubule generation. Downregulation in *MAPT*^*P301S*^;*Tyrobp*^*-/-*^ mice could further decrease the microtubule density and axonal integrity that are already altered in mouse models of tauopathy [40, 40–42]. Among the upregulated DEGs, *Zfp97* (logFC = 0.7) is also upregulated in Huntington’s disease [43] and *Kirrel3* (logFC = 0.3) has been suggested as a potential risk gene for AD [44].

## Discussion

In summary, using a variety of in vivo mouse model systems, we have observed (1) that a deficiency of TYROBP in microglia is associated with increased phospho-tau stoichiometry and accelerated spread of tauopathy in mouse models; (2) that the deletion of *Tyrobp* induces a decrease of C1q, which may underlie the beneficial effects on memory and synaptic plasticity; (3) that TREM2 deficiency leads to an increased phosphorylation and diffusion of tau but without alteration of the complement cascade.

The key observation in the current study is that phenotypic and pathophysiological symptoms and signs of tauopathy toxicity can be relieved when tauopathy model mice are constitutively deficient in a microglial-/dendritic cell-specific molecule (TYROBP), despite the fact that the severity of the tauopathy is not abated. Along with others, we recently identified *TYROBP* as a candidate LOAD hub/driver [2]. In AD amyloidosis mouse models, absence of TYROBP decreases recruitment of microglia around amyloid plaques [10, 11]. This altered accumulation of plaque-associated microglia has been proposed to lead to impairment of the compaction of amyloid deposits [10, 11], but this is the first study examining the relationship between TYROBP and tau.

Several studies have suggested that microglia might play significant roles not only in pathophysiology of amyloidosis but also in the pathophysiology of tauopathy [12, 13, 45]. In addition to the central involvement of microglia in inflammation and cytokine production, data indicate that reactive microglia can enhance tau pathology by promoting tauopathy spread. The first study along this line provided evidence that activated microglia can participate in both the induction of tau pathology and in its subsequent transneuronal spread [13]. Another study provided further evidence that microglia can promote tau dissemination and that depleting microglia suppresses tauopathy propagation [12]. In the face of these precedents, our report that *Tyrobp* deletion promotes tauopathy spread and tau phosphorylation is especially surprising because these biomarkers that are usually associated with disease progression develop in the face of apparent reduction in phenotypic and functional clinical features. Among a variety of propagation mechanisms such as transneuronal and trans-synaptic diffusions, tau also spreads via the extracellular space [46, 47]. Moreover, TREM2 and TYROBP play critical roles in the formation of the microglia barrier around amyloid plaques [11]. Thus, our current results suggest that TYROBP may play a specific role in detection by microglia of the presence of free, soluble tau in brain interstices and thereby plays a role in limiting the diffusion of tau through the brain. This could potentially explain our observation of increased tau spread in the presence of decreased microgliosis.

Another unexpected biomarker observation that we report here is an increase in the levels of phospho-tau, again despite the improvement in clinical phenotype. Although this result is discrepant with many early observations on clinico-phosphorylation status correlation, there is at least one tau phosphorylation site located at residue Thr^205^ and recognized by antibody AT8, for which increased stoichiometry of phosphorylation exerts a beneficial/protective effect in the prevention or neutralization of Aβ toxicity [48]. The other potential phospho-acceptor sites typically associated with tau hyperphosphorylation and toxicity include Ser^9^, Ser^26^, Thr^175^, Ser^199^, Thr^231^, Ser^262^, Ser^356^ and Ser^396^ [49–51]. Ser^396^ was also increased by TYROBP deficiency, suggesting that multiple competing effects are occurring simultaneously, indicating that the beneficial effects are apparently sufficiently robust to overcome the competing detrimental effects.

What are the molecular, cellular and subcellular underpinnings of the collection of apparently disparate features of the *MAPT*^*P301S*^;*Tyrobp*^*-/-*^ mice? One of the most puzzling set of traits is the paradoxical clinical and electrophysiological benefit despite an increase in tauopathy spread. It is indeed intriguing that a deficiency of TYROBP leading to an altered microglia barrier increases tau phosphorylation and spread while it has been previously reported that depletion of microglia dramatically suppresses the propagation of tau and that reactive microglia drive tau spreading [12, 13].

It is also logical to compare the effects of TYROBP or TREM2 deficiency on tauopathy, although this is somewhat difficult due to discrepant findings reported to date from *Trem2* KOs crossed with mouse models of tauopathy [32, 33]. The failure to decrease tau phosphorylation, however, is present in all studies so far, as is the decrease in several aspects of microglial activation compared with tauopathy alone. Electrophysiological and behavior assays were not performed in these other cited studies. Despite the fact that similar results can be obtained when tauopathy is modulated with either a deficiency of TYROBP or a deficiency of TREM2, a possible explanation for the improvement in behavior and synaptic plasticity is the alteration of the complement system, which is disrupted by *Tyrobp* deletion, but in our study, not by *Trem2* deletion, contrary to the report by Leyns et al. [32]. Specifically, C1q, the initiating protein in the classical complement cascade, is mainly produced by microglia in the brain and dramatically increased during normal aging [27, 29]. One model that has been proposed posits that C1q and the complement system mediate synapse elimination [20]. Moreover, this synapse pruning has been shown to be inappropriately activated in AD [52]. In this regard, it is important to note that C1q is part of the complement subnetwork driven by *TYROBP* [2], but importantly, reversal of the subnetwork was not detectable by RNA-seq as performed in this study of tauopathy, a result that is different from our observations in *APP/PSEN1* mice deficient for TYROBP [53]. Considering the higher number of DEGs observed in *APP/PSEN1* mice (181 versus WT mice) compared with the 14 DEGs observed in *MAPT*^*P301S*^ mice (versus WT mice, FDR ≤ 0.1 in this article versus 0.05 in Haure-Mirande et al. [53]) and considering that the complement system is one of the top pathways disturbed in *APP/PSEN1* mice but not *MAPT*^*P301S*^ mice, one possible conclusion is that the amyloidogenic context is the key feature of AD that underlies the complement system perturbation. Therefore, although the improvement of the behavior and the synaptic plasticity seem to be mainly due to a transcriptomic restoration in *APP/PSEN1* mice, which appears not to be the case in *MAPT*^*P301S*^ mice. Further investigations of clinico-molecular and clinico-pathological correlations will be required in order to determine what underlies the “improved function yet worsening biomarkers” situation that occurs in TYROBP-deficient *MAPT*^*P301S*^ tauopathy mice.

## Electronic supplementary material

Supplementary legends

Supplementary Figure 1

Supplementary Figure 2

Supplementary Figure 3

Supplementary Figure 4

Supplementary Figure 5

Supplementary Table 1
