# Supplementary material for: Integrative approach to sporadic Alzheimer’s disease: deficiency of TYROBP in a tauopathy mouse model reduces C1q and normalizes clinical phenotype while increasing spread and state of phosphorylation of tau
Source: Mol Psychiatry. 2018 Oct 3;24(9):1383–97. doi: 10.1038/s41380-018-0258-3 (PMC6447470; doi:10.1038/s41380-018-0258-3)
Supplement: Supplementary file 1 — Supplementary legends [file 41380_2018_258_MOESM1_ESM.docx]

**Legends for Supplementary Figures and Table**

**Supplementary Figure 1: Microgliosis is associated with tau pathology in *MAPT^P301S^* mice.**

a. Representative images of anti-Iba1 immunohistochemistry in WT and *MAPT^P301S^* mice at 2 months of age, showing increased microglial reactivity (as indicated by Iba1-like immunoreactivity) in the brain of the tau transgenic mouse as compared to that of the wildtype mouse (WT).

b. Representative images of anti-Iba1 immunohistochemistry (top panel) and AT8 antibody immunohistochemistry (bottom panel) showing evidence that identical brain regions strongly positive for AT8 are also strongly positive for reactive microglia, including the hippocampus and piriform cortex *MAPT^P301S^ mice* (7 months of age).

**Supplementary Figure 2: Full-length western blots presented as cropped in main figures.**

**Supplementary Figure 3: Experimental paradigm to study spread of tau in the granular layer of the dentate gyrus after injection of AAV-tau-GFP in the medial entorhinal cortex.**

AAV-tau-GFP was injected in the medial entorhinal cortex to study the spread of tau-GFP from this structure to the hippocampus.

LEFT: Boxed area of interest shown with Nissl stain, corresponding to higher magnification fluorescent images (RIGHT). CA1sp: CA1, pyramidal layer; CA1sr: CA1, stratum radiatum; S: Subiculum; Ent: Entorhinal cortex; Hp: Hippocampus; Ctx: Cortex; Str: Striatum; PP: Perforant Pathway.

**Supplementary Figure 4: TREM2 deficiency leads to increased levels of phospho-tau in *MAPT^P301S^* mice at an older age than when increased levels of phospho-tau are evident in TYROBP deficient mice; C1q RNA and protein are not obviously affected by TREM2 deficiency.**

a. Western blots of phosphorylated tau (pS202/pT205) and total tau in *MAPT^P301S^* mice with and without deletion of *Tyrobp* or *Trem2* at 4 months of age.

b. Densitometric analysis of western blot shown in A (n = 4-7 mice per groups). Error bars represent means ± SEM. Statistical analyses were performed using a one-way ANOVA followed by Tukey’s post-hoc test (B) or a Student’s t-test (E), *p<0.05; ***p<0.001.

c. Representative images of immunohistochemistry using an antibody that detects phosphorylated tau (pS396) to study *MAPT^P301S^* mice with and without deletion of *Tyrobp* or *Trem2* (4 months of age).

d. Representative images of immunohistochemistry using antibodies that detect phosphorylated tau (pS202/pT205 and pS396) to study *MAPT^P301S^* mice with and without deletion of *Trem2* (8 months of age).

e. RT-qPCR analysis for *TREM2* and *C1q* in *MAPT^P301S^* mice with and without knockout of *Trem2*. Analysis was performed on prefrontal cortex samples from mice sacrified at 8 months of age

f. Western blot of C1q and GAPDH on cortical samples from mice sacrified at 8 months of age (n = 3 per group).

g. TREM2 targeting strategy used to generate the *Trem2^-/-^* mice.

**Supplementary Figure 5: Differential gene expression analysis**

a. RNA sequencing analysis of prefrontal cortex in *MAPT^P301S^*, *MAPT^P301S^;Tyrobp ^-/-^* and WT mice (11 months of age; n = 4 samples per group). The small number of reads mapped to the human *MAPT* gene in WT mice is likely due to similarities with the mouse gene.

b. Number of up- and down-regulated genes in the same groups as (a) (FDR ≤ 0.1; FC ≥ 1.2).

c. Volcano plot representations of the differentially expressed genes (DEGs). Red dots indicate significant DEGs.

d-f. Top 10 DEGs up- and down-regulated in *MAPT^P301S^;Tyrobp ^-/-^* vs *MAPT^P301S^* (d), *MAPT^P301S^;Tyrobp ^-/-^* vs WT (e) and *MAPT^P301S^* vs WT (f).

g. Overlap among the different sets of DEGs.

Differential gene expression threshold was set at change ≥1.2 and adjusted p value < 0.1. RNA sequencing was performed on a total of 12 prefrontal cortex samples. Only females (all genotype groups) were used for this study.

**Supplementary Table 1: List of differentially expressed genes (DEGs) with adjusted p value (FDR)<0.1 and fold change (FC)>1.2**
