# Supplementary figures and images for: Integrative approach to sporadic Alzheimer’s disease: deficiency of TYROBP in a tauopathy mouse model reduces C1q and normalizes clinical phenotype while increasing spread and state of phosphorylation of tau
Source: Mol Psychiatry. 2018 Oct 3;24(9):1383–97. doi: 10.1038/s41380-018-0258-3 (PMC6447470; doi:10.1038/s41380-018-0258-3)

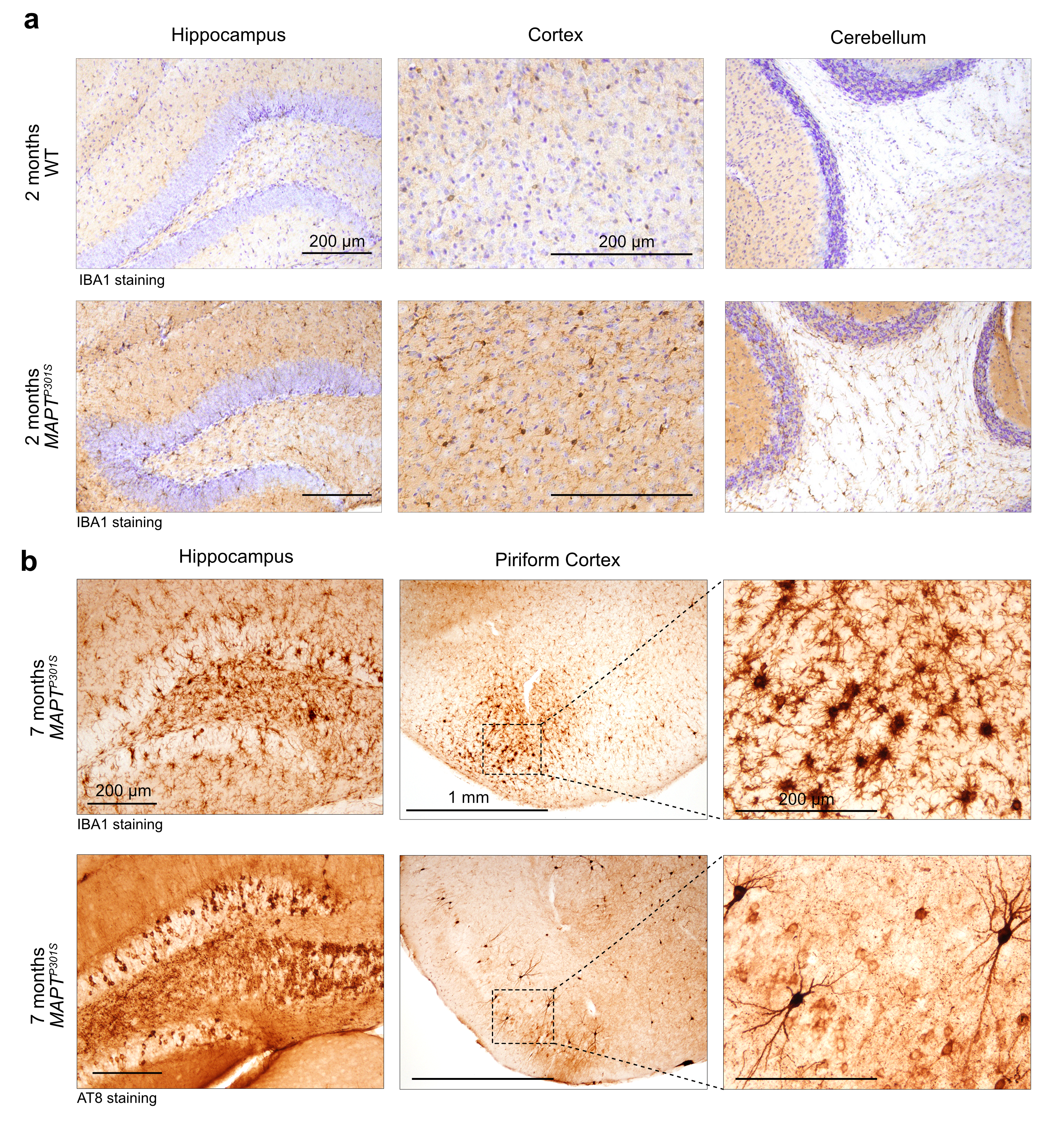

Supplement: Supplementary file 2 — Supplementary Figure 1 [file 41380_2018_258_MOESM2_ESM.tif]

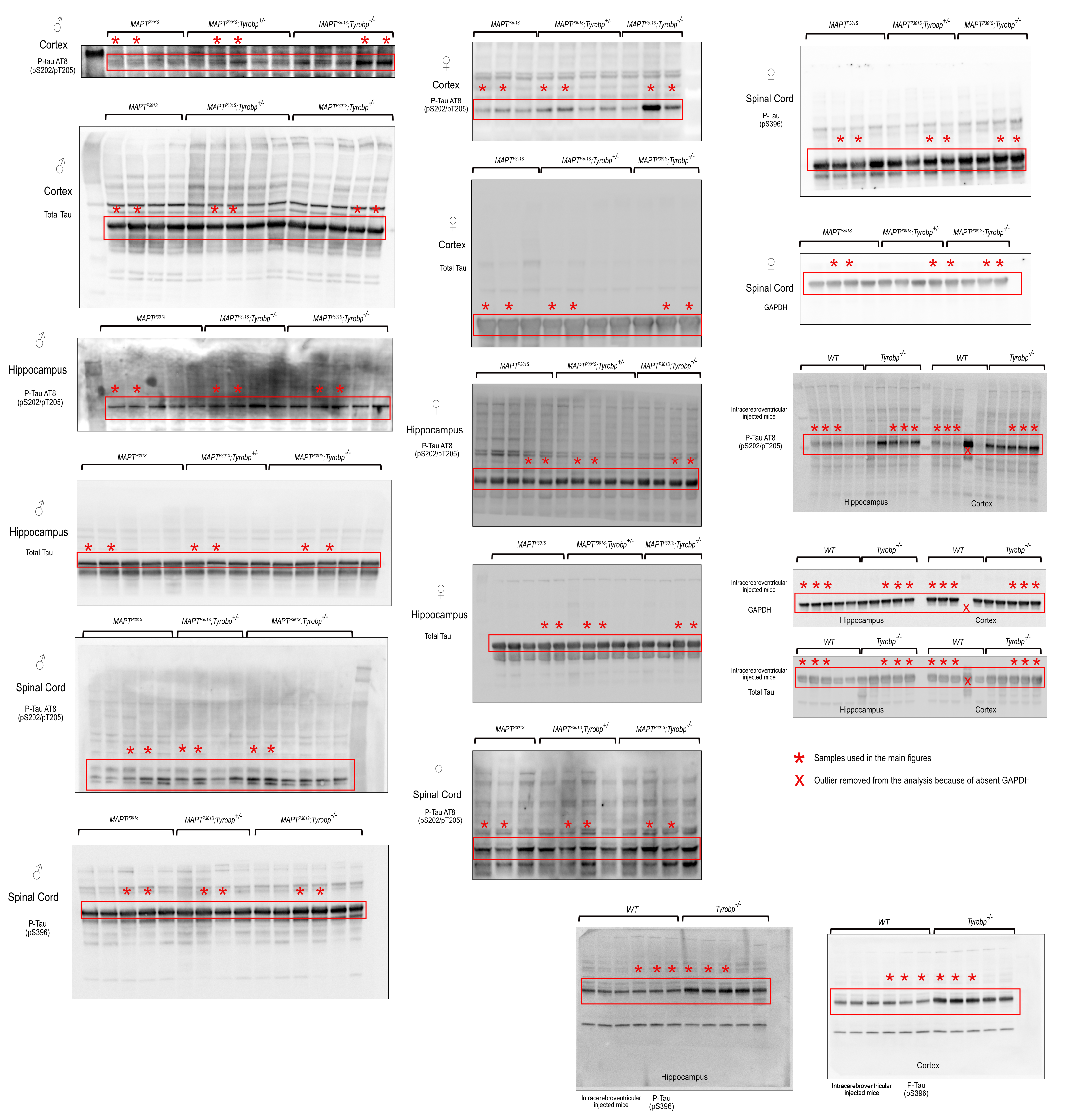

Supplement: Supplementary file 3 — Supplementary Figure 2 [file 41380_2018_258_MOESM3_ESM.tif]

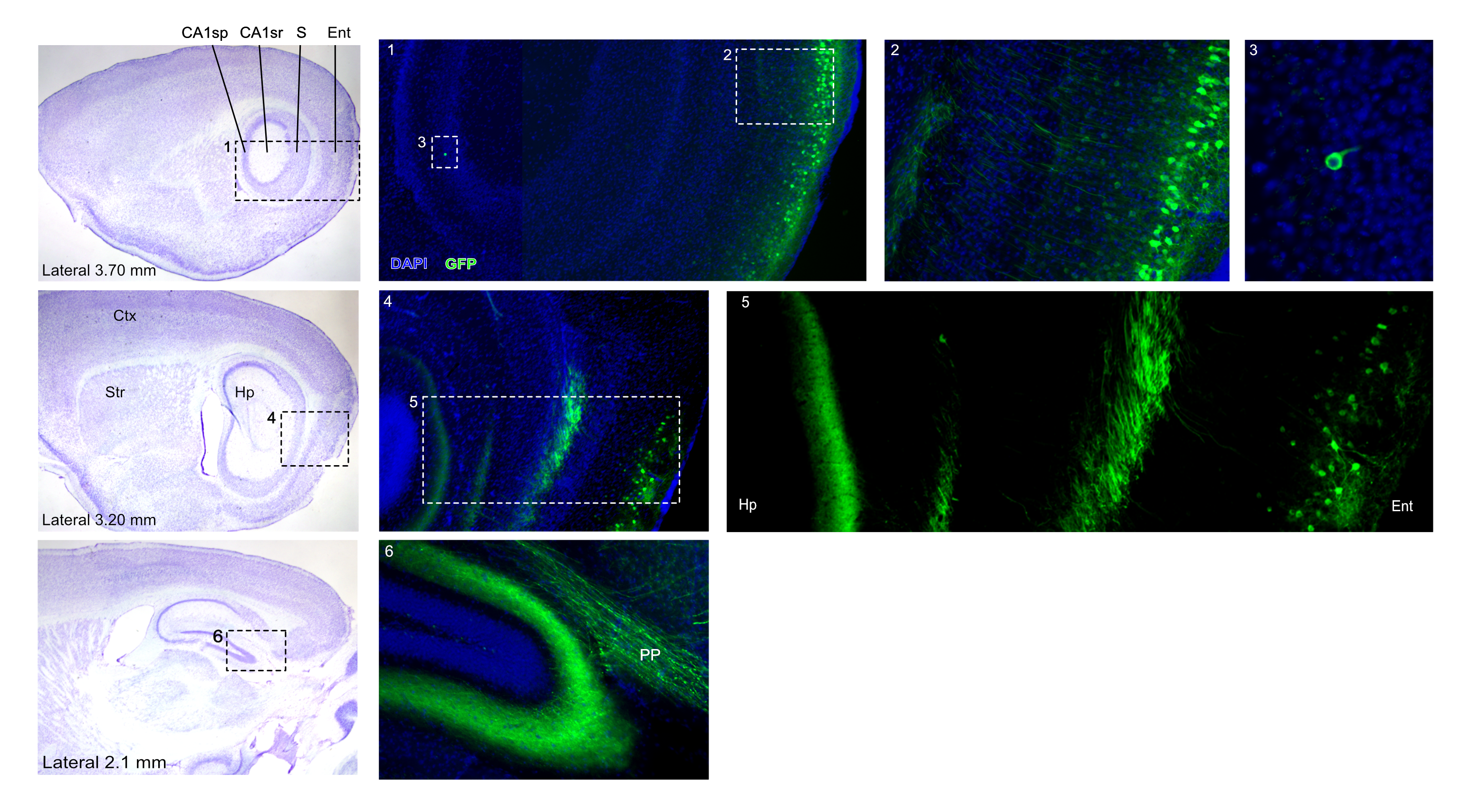

Supplement: Supplementary file 4 — Supplementary Figure 3 [file 41380_2018_258_MOESM4_ESM.tif]

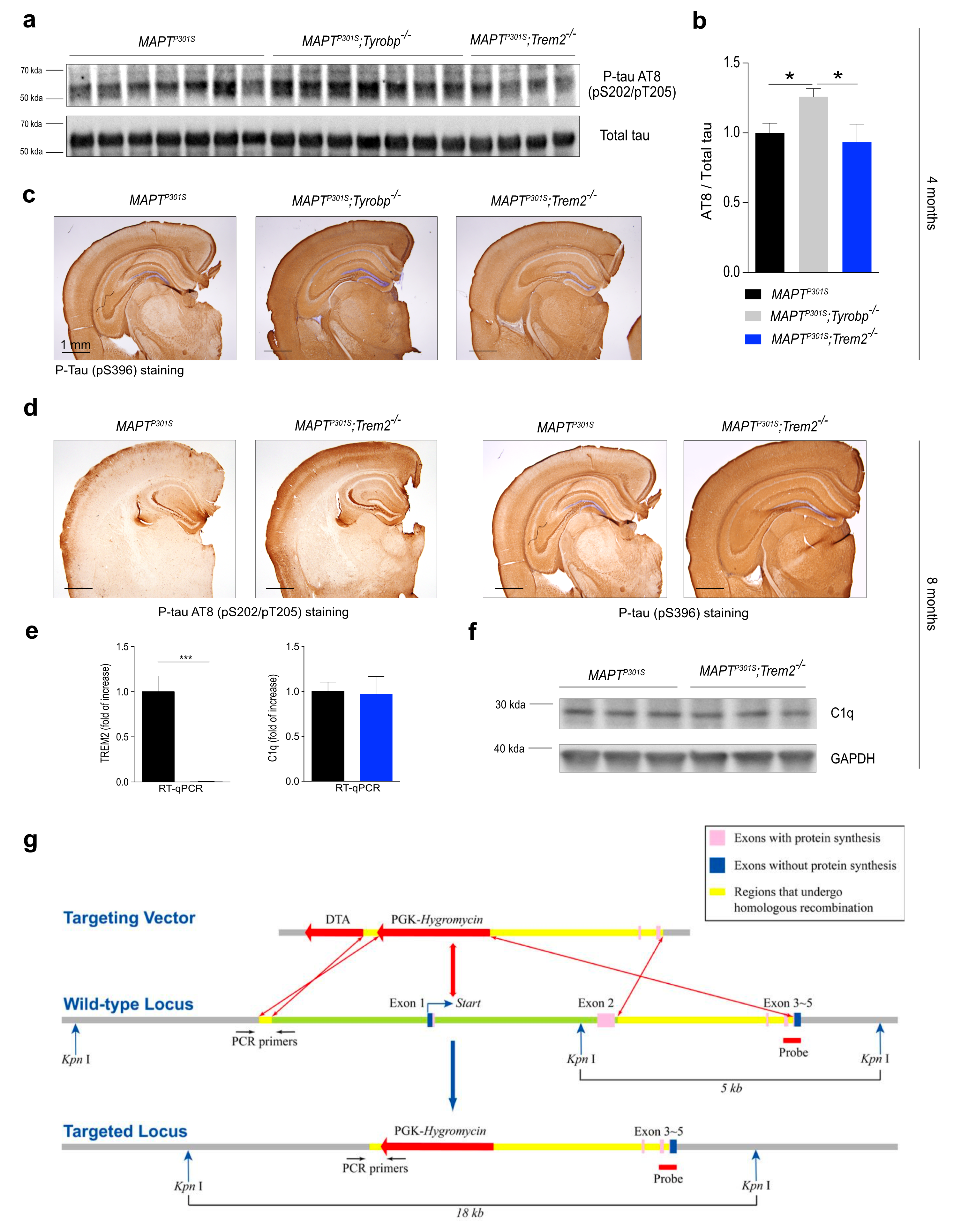

Supplement: Supplementary file 5 — Supplementary Figure 4 [file 41380_2018_258_MOESM5_ESM.tif]

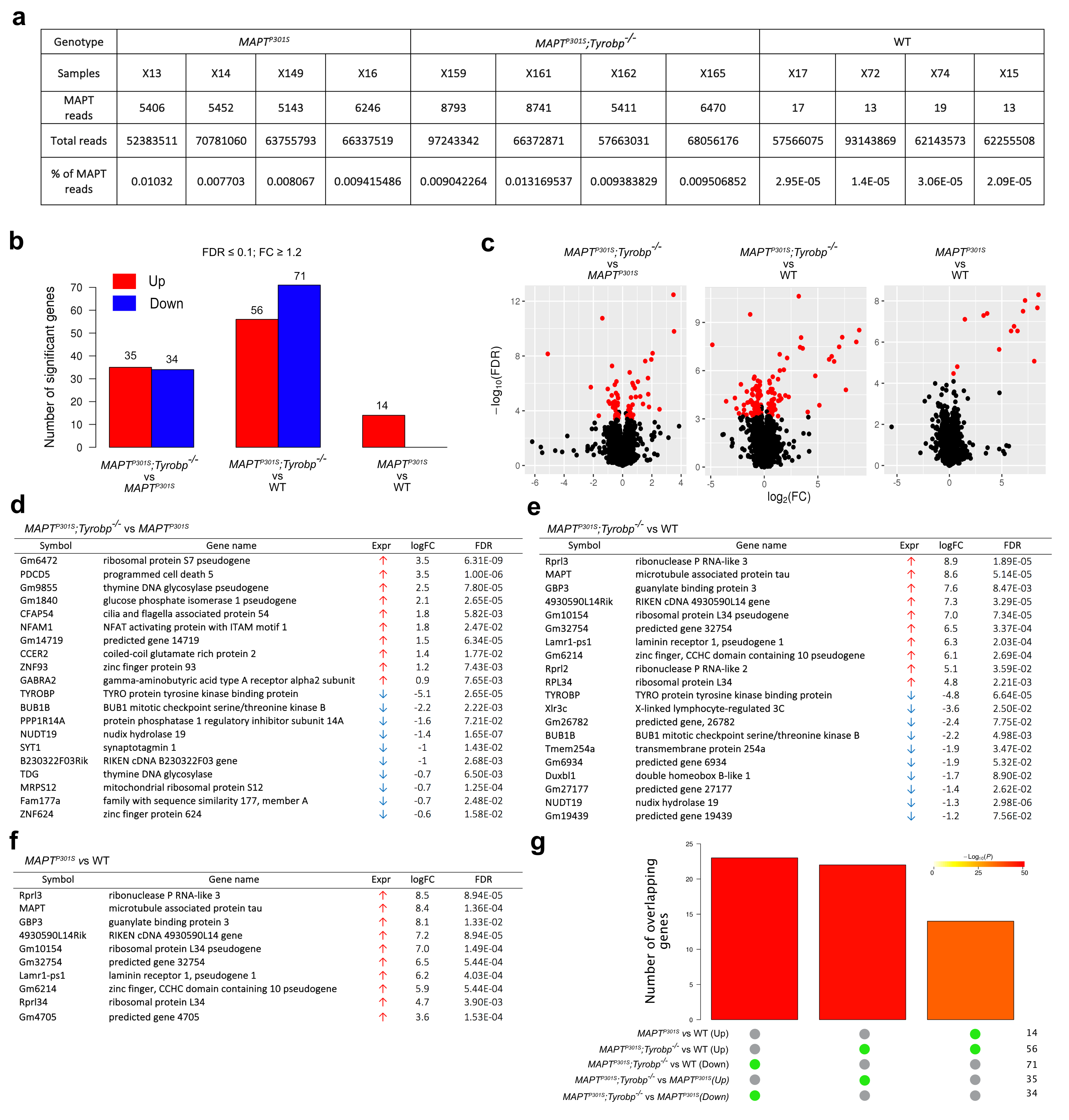

Supplement: Supplementary file 6 — Supplementary Figure 5 [file 41380_2018_258_MOESM6_ESM.tif]
