# Supplementary material for: Integrative approach to sporadic Alzheimer’s disease: deficiency of TYROBP in a tauopathy mouse model reduces C1q and normalizes clinical phenotype while increasing spread and state of phosphorylation of tau
Source: Mol Psychiatry. 2018 Oct 3;24(9):1383–97. doi: 10.1038/s41380-018-0258-3 (PMC6447470; doi:10.1038/s41380-018-0258-3)
Supplement: Supplementary file 7 — Supplementary Table 1 [file 41380_2018_258_MOESM7_ESM.pdf]

Geneid	Symbol	human_gene	logFC	AveExpr	t	P.Value	adj.P.Val	Contrast
ENSMUSG00000095597	Gm6472	NA	3.5	1.5	31.7	3.35E-13	6.31E-09	MAPTP301S X Tyrobp -/- vs MAPTP301S
ENSMUSG00000034875	Nudt19	NUDT19	-1.4	5.5	-22.9	1.75E-11	1.65E-07	MAPTP301S X Tyrobp -/- vs MAPTP301S
ENSMUSG00000102824	Pdcd5-ps	PDCD5	3.5	0.0	19.1	1.60E-10	1.00E-06	MAPTP301S X Tyrobp -/- vs MAPTP301S
ENSMUSG00000043192	Gm1840	NA	2.1	1.0	14.0	6.34E-09	2.65E-05	MAPTP301S X Tyrobp -/- vs MAPTP301S
ENSMUSG00000030579	Tyrobp	TYROBP	-5.1	1.5	-13.8	7.03E-09	2.65E-05	MAPTP301S X Tyrobp -/- vs MAPTP301S
ENSMUSG00000109205	Gm44954	NA	2.0	0.1	12.8	1.80E-08	5.64E-05	MAPTP301S X Tyrobp -/- vs MAPTP301S
ENSMUSG00000082192	Gm14719	NA	1.5	0.2	12.5	2.36E-08	6.34E-05	MAPTP301S X Tyrobp -/- vs MAPTP301S
ENSMUSG00000045948	Mrps12	MRPS12	-0.7	4.3	-11.6	5.35E-08	0.000125856	MAPTP301S X Tyrobp -/- vs MAPTP301S
ENSMUSG00000021510	Zfp729a	ZNF729	0.5	4.7	10.5	1.58E-07	0.000331589	MAPTP301S X Tyrobp -/- vs MAPTP301S
ENSMUSG00000114442	AC159264.1	NA	1.7	0.9	9.7	4.14E-07	0.000779052	MAPTP301S X Tyrobp -/- vs MAPTP301S
ENSMUSG00000044573	Acp1	ACP1	-0.4	5.6	-9.2	7.29E-07	0.00117601	MAPTP301S X Tyrobp -/- vs MAPTP301S
ENSMUSG00000113047	CT009718.3	NA	1.0	1.8	9.2	7.49E-07	0.00117601	MAPTP301S X Tyrobp -/- vs MAPTP301S
ENSMUSG00000080893	Gm15920	NA	0.7	4.8	9.0	9.51E-07	0.001377515	MAPTP301S X Tyrobp -/- vs MAPTP301S
ENSMUSG00000042831	Alkbh6	ALKBH6	-0.5	4.1	-8.7	1.25E-06	0.001684288	MAPTP301S X Tyrobp -/- vs MAPTP301S
ENSMUSG00000040466	Blvrb	BLVRB	0.7	2.7	8.6	1.40E-06	0.001757287	MAPTP301S X Tyrobp -/- vs MAPTP301S
ENSMUSG00000040084	Bub1b	BUB1B	-2.2	0.7	-8.4	1.89E-06	0.002226416	MAPTP301S X Tyrobp -/- vs MAPTP301S
ENSMUSG00000053985	Zfp14	ZFP14	0.8	3.7	8.3	2.19E-06	0.002427098	MAPTP301S X Tyrobp -/- vs MAPTP301S
ENSMUSG00000097509	B230322F03Rik	NA	-1.0	2.2	-8.2	2.57E-06	0.002683987	MAPTP301S X Tyrobp -/- vs MAPTP301S
ENSMUSG00000063808	Gpatch1	GPATCH1	-0.5	4.2	-7.6	5.08E-06	0.005033166	MAPTP301S X Tyrobp -/- vs MAPTP301S
ENSMUSG00000020014	Cfap54	CFAP54	1.8	4.6	7.5	6.19E-06	0.005826319	MAPTP301S X Tyrobp -/- vs MAPTP301S
ENSMUSG00000021476	Habp4	HABP4	-0.4	5.6	-7.4	7.16E-06	0.006423816	MAPTP301S X Tyrobp -/- vs MAPTP301S
ENSMUSG00000034674	Tdg	TDG	-0.7	4.6	-7.3	7.59E-06	0.00650128	MAPTP301S X Tyrobp -/- vs MAPTP301S
ENSMUSG00000055341	Zfp457	ZNF93	1.2	0.3	7.2	9.07E-06	0.007430562	MAPTP301S X Tyrobp -/- vs MAPTP301S
ENSMUSG00000000560	Gabra2	GABRA2	0.9	5.8	7.2	9.76E-06	0.007658133	MAPTP301S X Tyrobp -/- vs MAPTP301S
ENSMUSG00000020019	Ntn4	NTN4	0.6	3.4	7.1	1.07E-05	0.008089035	MAPTP301S X Tyrobp -/- vs MAPTP301S
ENSMUSG00000055480	Zfp458	ZNF43	-0.3	4.6	-7.0	1.17E-05	0.008488673	MAPTP301S X Tyrobp -/- vs MAPTP301S
ENSMUSG00000058402	Zfp420	ZNF420	-0.5	4.2	-6.6	2.11E-05	0.014382226	MAPTP301S X Tyrobp -/- vs MAPTP301S
ENSMUSG00000035864	Syt1	SYT1	-1.0	9.2	-6.6	2.14E-05	0.014382226	MAPTP301S X Tyrobp -/- vs MAPTP301S
ENSMUSG00000094622	Gm3055	ZNF624	-0.6	3.0	-6.5	2.45E-05	0.01589214	MAPTP301S X Tyrobp -/- vs MAPTP301S
ENSMUSG00000093571	Gm24757	NA	-0.7	1.7	-6.5	2.59E-05	0.01626796	MAPTP301S X Tyrobp -/- vs MAPTP301S
ENSMUSG00000094002	Gm9866	NA	-0.3	5.1	-6.5	2.71E-05	0.016479038	MAPTP301S X Tyrobp -/- vs MAPTP301S
ENSMUSG00000112640	AC172027.3	NA	-0.9	2.3	-6.4	3.01E-05	0.017724694	MAPTP301S X Tyrobp -/- vs MAPTP301S
ENSMUSG00000053898	Ech1	ECH1	-0.5	5.1	-6.4	3.12E-05	0.017724694	MAPTP301S X Tyrobp -/- vs MAPTP301S
ENSMUSG00000096257	Ccer2	CCER2	1.4	-0.3	6.4	3.20E-05	0.017724694	MAPTP301S X Tyrobp -/- vs MAPTP301S
ENSMUSG00000095990	Zfp97	NA	0.7	3.0	6.3	3.61E-05	0.019310603	MAPTP301S X Tyrobp -/- vs MAPTP301S
ENSMUSG00000058246	Gm10037	NA	-0.6	1.9	-6.3	3.69E-05	0.019310603	MAPTP301S X Tyrobp -/- vs MAPTP301S
ENSMUSG00000030417	Pdcd5	PDCD5	-0.3	4.5	-6.2	3.92E-05	0.01996634	MAPTP301S X Tyrobp -/- vs MAPTP301S
ENSMUSG00000040424	Hipk4	HIPK4	-0.6	4.4	-6.1	4.56E-05	0.022531613	MAPTP301S X Tyrobp -/- vs MAPTP301S
ENSMUSG00000059975	Zfp74	ZNF569	0.4	3.7	6.1	4.67E-05	0.022531613	MAPTP301S X Tyrobp -/- vs MAPTP301S
ENSMUSG00000058099	Nfam1	NFAM1	1.8	-0.1	6.0	5.26E-05	0.024760889	MAPTP301S X Tyrobp -/- vs MAPTP301S
ENSMUSG00000021594	Srd5a1	SRD5A1	-0.5	1.7	-6.0	5.39E-05	0.024764232	MAPTP301S X Tyrobp -/- vs MAPTP301S
ENSMUSG00000094103	1700047I17Rik2	NA	-0.7	4.9	-6.0	5.60E-05	0.024856648	MAPTP301S X Tyrobp -/- vs MAPTP301S
ENSMUSG00000030584	Dpf1	DPF1	-0.5	4.1	-6.0	5.68E-05	0.024856648	MAPTP301S X Tyrobp -/- vs MAPTP301S
ENSMUSG00000022899	Slc15a2	SLC15A2	0.5	4.9	5.9	6.65E-05	0.028474184	MAPTP301S X Tyrobp -/- vs MAPTP301S
ENSMUSG00000096718	Zfp781	NA	-0.5	3.8	-5.8	7.41E-05	0.031020428	MAPTP301S X Tyrobp -/- vs MAPTP301S
ENSMUSG00000085666	Gm9855	NA	2.5	0.0	5.8	7.80E-05	0.031934108	MAPTP301S X Tyrobp -/- vs MAPTP301S
ENSMUSG00000073427	Gm4924	NA	0.5	3.8	5.8	8.17E-05	0.032722736	MAPTP301S X Tyrobp -/- vs MAPTP301S
ENSMUSG00000021597	Slf1	SLF1	0.4	6.0	5.7	8.94E-05	0.035059597	MAPTP301S X Tyrobp -/- vs MAPTP301S
ENSMUSG00000030589	Rasgrp4	RASGRP4	-0.5	2.6	-5.7	9.44E-05	0.036283133	MAPTP301S X Tyrobp -/- vs MAPTP301S
ENSMUSG00000090667	Gm765	NA	0.5	3.1	5.4	0.000139266	0.051427508	MAPTP301S X Tyrobp -/- vs MAPTP301S
ENSMUSG00000030590	Fam98c	FAM98C	-0.4	3.4	-5.3	0.000160401	0.05594118	MAPTP301S X Tyrobp -/- vs MAPTP301S
ENSMUSG00000045092	S1pr1	S1PR1	-0.4	6.3	-5.3	0.000182344	0.06243789	MAPTP301S X Tyrobp -/- vs MAPTP301S
ENSMUSG00000020890	Gucy2e	GUCY2D	0.7	0.5	5.2	0.00019916	0.065728066	MAPTP301S X Tyrobp -/- vs MAPTP301S
ENSMUSG00000078762	Haus5	HAUS5	-0.5	2.1	-5.2	0.000201577	0.065728066	MAPTP301S X Tyrobp -/- vs MAPTP301S
ENSMUSG00000069206	Zfp874a	NA	-0.3	4.3	-5.2	0.000202423	0.065728066	MAPTP301S X Tyrobp -/- vs MAPTP301S
ENSMUSG00000040390	Map3k10	MAP3K10	0.4	5.4	5.2	0.000215903	0.068917117	MAPTP301S X Tyrobp -/- vs MAPTP301S
ENSMUSG00000037166	Ppp1r14a	PPP1R14A	-1.6	-0.1	-5.1	0.000229754	0.07211592	MAPTP301S X Tyrobp -/- vs MAPTP301S
ENSMUSG00000034867	Ankrd27	ANKRD27	0.3	5.0	5.1	0.000235089	0.072580953	MAPTP301S X Tyrobp -/- vs MAPTP301S
ENSMUSG00000020018	Snrpf	SNRPF	-0.5	3.4	-5.1	0.000244916	0.073766038	MAPTP301S X Tyrobp -/- vs MAPTP301S
ENSMUSG00000039994	Timeless	TIMELESS	0.6	2.5	5.1	0.000246762	0.073766038	MAPTP301S X Tyrobp -/- vs MAPTP301S

ENSMUSG00000032036	Kirrel3	KIRREL3	0.3	4.5	5.1	0.000255675	0.074350282	MAPTP301S X Tyrobp -/- vs MAPTP301S
ENSMUSG00000084347	Akt2-ps	NA	0.6	2.1	5.1	0.000256612	0.074350282	MAPTP301S X Tyrobp -/- vs MAPTP301S
ENSMUSG00000113536	CT030170.4	NA	0.5	2.4	5.0	0.000268073	0.076082009	MAPTP301S X Tyrobp -/- vs MAPTP301S
ENSMUSG00000098891	Gm27753	NA	0.5	2.0	5.0	0.000270668	0.076082009	MAPTP301S X Tyrobp -/- vs MAPTP301S
ENSMUSG00000107092	Gm7993	NA	0.6	1.7	5.0	0.000289892	0.080287413	MAPTP301S X Tyrobp -/- vs MAPTP301S
ENSMUSG00000036915	Kirrel2	KIRREL2	0.5	2.9	5.0	0.000298417	0.08145048	MAPTP301S X Tyrobp -/- vs MAPTP301S
ENSMUSG00000085105	Gm12758	NA	-0.3	3.4	-4.9	0.000343375	0.092382629	MAPTP301S X Tyrobp -/- vs MAPTP301S
ENSMUSG00000110686	Gm45855	NA	0.6	2.3	4.8	0.000369267	0.097949313	MAPTP301S X Tyrobp -/- vs MAPTP301S
ENSMUSG00000036168	Ccdc38	CCDC38	-0.5	2.8	-4.8	0.000380478	0.099521442	MAPTP301S X Tyrobp -/- vs MAPTP301S
ENSMUSG00000088025	Rprl3	NA	8.5	5.6	14.2	4.99E-09	8.93E-05	MAPTP301S vs WT
ENSMUSG00000106540	4930590L14Rik	NA	7.2	-2.1	13.5	9.49E-09	8.93E-05	MAPTP301S vs WT
ENSG00000186868	human_Tau	MAPT	8.4	3.7	12.6	2.16E-08	0.000135785	MAPTP301S vs WT
ENSMUSG00000066116	Gm10154	NA	7.0	0.2	12.1	3.17E-08	0.000149054	MAPTP301S vs WT
ENSMUSG00000093686	Gm4705	NA	3.6	0.0	11.9	4.07E-08	0.000153472	MAPTP301S vs WT
ENSMUSG00000094685	Gm5900	NA	3.3	-0.5	11.6	5.10E-08	0.000160181	MAPTP301S vs WT
ENSMUSG00000028028	Alpk1	ALPK1	1.5	3.5	11.2	7.81E-08	0.000210128	MAPTP301S vs WT
ENSMUSG00000081229	Lamr1-ps1	NA	6.2	-2.1	10.5	1.71E-07	0.000403315	MAPTP301S vs WT
ENSMUSG00000104544	Gm32754	NA	6.5	-2.6	10.0	2.88E-07	0.000544555	MAPTP301S vs WT
ENSMUSG00000095465	Gm6214	NA	5.9	-2.3	10.0	2.89E-07	0.000544555	MAPTP301S vs WT
ENSMUSG00000061669	Rpl34-ps2	RPL34	4.7	-1.7	8.2	2.28E-06	0.003900847	MAPTP301S vs WT
ENSMUSG00000040264	Gbp2b	GBP3	8.1	-1.5	7.3	8.48E-06	0.013312507	MAPTP301S vs WT
ENSMUSG00000068396	Rpl34-ps1	NA	0.8	2.0	6.8	1.57E-05	0.022689944	MAPTP301S vs WT
ENSMUSG00000079037	Prnp	PRNP	0.4	9.5	6.3	3.37E-05	0.04527656	MAPTP301S vs WT
ENSMUSG00000095597	Gm6472	NA	3.2	1.5	22.4	2.35E-11	4.42E-07	MAPTP301S X Tyrobp -/- vs WT
ENSMUSG00000034875	Nudt19	NUDT19	-1.3	5.5	-18.0	3.17E-10	2.98E-06	MAPTP301S X Tyrobp -/- vs WT
ENSMUSG00000088025	Rprl3	NA	8.9	5.6	14.9	3.01E-09	1.89E-05	MAPTP301S X Tyrobp -/- vs WT
ENSMUSG00000106540	4930590L14Rik	NA	7.3	-2.1	13.6	8.36E-09	3.30E-05	MAPTP301S X Tyrobp -/- vs WT
ENSMUSG00000102824	Pdcd5-ps	PDCD5	3.4	0.0	13.6	8.75E-09	3.30E-05	MAPTP301S X Tyrobp -/- vs WT
ENSG00000186868	human_Tau	MAPT	8.6	3.7	12.9	1.64E-08	5.15E-05	MAPTP301S X Tyrobp -/- vs WT
ENSMUSG00000030579	Tyrobp	TYROBP	-4.8	1.5	-12.4	2.47E-08	6.64E-05	MAPTP301S X Tyrobp -/- vs WT
ENSMUSG00000066116	Gm10154	NA	7.0	0.2	12.1	3.32E-08	7.34E-05	MAPTP301S X Tyrobp -/- vs WT
ENSMUSG00000094685	Gm5900	NA	3.3	-0.5	12.0	3.51E-08	7.34E-05	MAPTP301S X Tyrobp -/- vs WT
ENSMUSG00000093686	Gm4705	NA	3.6	0.0	11.9	4.13E-08	7.78E-05	MAPTP301S X Tyrobp -/- vs WT
ENSMUSG00000028028	Alpk1	ALPK1	1.4	3.5	11.0	9.67E-08	0.000165515	MAPTP301S X Tyrobp -/- vs WT
ENSMUSG00000081229	Lamr1-ps1	NA	6.3	-2.1	10.7	1.30E-07	0.000203941	MAPTP301S X Tyrobp -/- vs WT
ENSMUSG00000043192	Gm1840	NA	2.1	1.0	10.5	1.64E-07	0.00023734	MAPTP301S X Tyrobp -/- vs WT
ENSMUSG00000095465	Gm6214	NA	6.1	-2.3	10.3	2.00E-07	0.000269157	MAPTP301S X Tyrobp -/- vs WT
ENSMUSG00000104544	Gm32754	NA	6.5	-2.6	10.0	2.69E-07	0.000337126	MAPTP301S X Tyrobp -/- vs WT
ENSMUSG00000109205	Gm44954	NA	1.8	0.1	9.0	8.90E-07	0.00104726	MAPTP301S X Tyrobp -/- vs WT
ENSMUSG00000082192	Gm14719	NA	1.5	0.2	8.9	9.90E-07	0.001096326	MAPTP301S X Tyrobp -/- vs WT
ENSMUSG00000061669	Rpl34-ps2	RPL34	4.8	-1.7	8.3	2.12E-06	0.00221606	MAPTP301S X Tyrobp -/- vs WT
ENSMUSG00000080893	Gm15920	NA	0.8	4.8	8.2	2.41E-06	0.002297174	MAPTP301S X Tyrobp -/- vs WT
ENSMUSG00000094622	Gm3055	ZNF624	-0.9	3.0	-8.2	2.44E-06	0.002297174	MAPTP301S X Tyrobp -/- vs WT
ENSMUSG00000034674	Tdg	TDG	-1.0	4.6	-8.0	3.09E-06	0.002769564	MAPTP301S X Tyrobp -/- vs WT
ENSMUSG00000000560	Gabra2	GABRA2	1.3	5.8	7.9	3.41E-06	0.002923141	MAPTP301S X Tyrobp -/- vs WT
ENSMUSG00000045948	Mrps12	MRPS12	-0.6	4.3	-7.8	4.30E-06	0.003437568	MAPTP301S X Tyrobp -/- vs WT
ENSMUSG00000079037	Prnp	PRNP	0.5	9.5	7.8	4.38E-06	0.003437568	MAPTP301S X Tyrobp -/- vs WT
ENSMUSG00000055480	Zfp458	ZNF43	-0.4	4.6	-7.7	4.95E-06	0.003728941	MAPTP301S X Tyrobp -/- vs WT
ENSMUSG00000004558	Ndrg2	NDRG2	0.5	9.0	7.5	6.05E-06	0.004380066	MAPTP301S X Tyrobp -/- vs WT
ENSMUSG00000040084	Bub1b	BUB1B	-2.2	0.7	-7.4	7.14E-06	0.004982353	MAPTP301S X Tyrobp -/- vs WT
ENSMUSG00000044573	Acp1	ACP1	-0.4	5.6	-7.3	7.82E-06	0.005257715	MAPTP301S X Tyrobp -/- vs WT
ENSMUSG00000096718	Zfp781	NA	-0.7	3.8	-7.2	9.25E-06	0.006009421	MAPTP301S X Tyrobp -/- vs WT
ENSMUSG00000042831	Alkbh6	ALKBH6	-0.5	4.1	-7.1	1.03E-05	0.006481286	MAPTP301S X Tyrobp -/- vs WT
ENSMUSG00000092837	Rpph1	RPPH1	-0.9	12.0	-7.0	1.24E-05	0.007534402	MAPTP301S X Tyrobp -/- vs WT
ENSMUSG00000046688	Tifa	TIFA	0.6	3.3	6.9	1.40E-05	0.008177058	MAPTP301S X Tyrobp -/- vs WT
ENSMUSG00000040466	Blvrb	BLVRB	0.8	2.7	6.9	1.43E-05	0.008177058	MAPTP301S X Tyrobp -/- vs WT
ENSMUSG00000112640	AC172027.3	NA	-1.1	2.3	-6.9	1.50E-05	0.008325894	MAPTP301S X Tyrobp -/- vs WT
ENSMUSG00000040264	Gbp2b	GBP3	7.6	-1.5	6.8	1.58E-05	0.008479346	MAPTP301S X Tyrobp -/- vs WT
ENSMUSG00000045092	S1pr1	S1PR1	-0.7	6.3	-6.8	1.66E-05	0.008695233	MAPTP301S X Tyrobp -/- vs WT
ENSMUSG00000094002	Gm9866	NA	-0.4	5.1	-6.7	1.83E-05	0.009312257	MAPTP301S X Tyrobp -/- vs WT
ENSMUSG00000057836	Xlr3a	NA	-1.6	0.5	-6.7	2.01E-05	0.00994635	MAPTP301S X Tyrobp -/- vs WT

ENSMUSG00000021510	Zfp729a	ZNF729	0.4	4.7	6.6	2.22E-05	0.010705923	MAPTP301S X Tyrobp -/- vs WT
ENSMUSG00000068396	Rpl34-ps1	NA	0.7	2.0	6.6	2.32E-05	0.010918368	MAPTP301S X Tyrobp -/- vs WT
ENSMUSG00000060012	Kif13b	KIF13B	-0.4	5.0	-6.5	2.43E-05	0.0111529	MAPTP301S X Tyrobp -/- vs WT
ENSMUSG00000113047	CT009718.3	NA	1.0	1.8	6.5	2.62E-05	0.011766381	MAPTP301S X Tyrobp -/- vs WT
ENSMUSG00000035864	Syt1	SYT1	-1.1	9.2	-6.4	2.84E-05	0.012439247	MAPTP301S X Tyrobp -/- vs WT
ENSMUSG00000058402	Zfp420	ZNF420	-0.6	4.2	-6.4	3.14E-05	0.013447601	MAPTP301S X Tyrobp -/- vs WT
ENSMUSG00000076617	Ighm	NA	-1.1	4.4	-6.3	3.30E-05	0.013814925	MAPTP301S X Tyrobp -/- vs WT
ENSMUSG00000005871	Apc	APC	-0.3	9.0	-6.3	3.40E-05	0.01392109	MAPTP301S X Tyrobp -/- vs WT
ENSMUSG00000020014	Cfap54	CFAP54	2.0	4.6	6.3	3.53E-05	0.013975042	MAPTP301S X Tyrobp -/- vs WT
ENSMUSG00000114442	AC159264.1	NA	1.4	0.9	6.3	3.56E-05	0.013975042	MAPTP301S X Tyrobp -/- vs WT
ENSMUSG00000069206	Zfp874a	NA	-0.4	4.3	-6.3	3.71E-05	0.014257177	MAPTP301S X Tyrobp -/- vs WT
ENSMUSG00000021594	Srd5a1	SRD5A1	-0.7	1.7	-6.2	3.84E-05	0.014480282	MAPTP301S X Tyrobp -/- vs WT
ENSMUSG00000091020	Gm5828	NA	2.3	-1.1	6.2	4.33E-05	0.015983027	MAPTP301S X Tyrobp -/- vs WT
ENSMUSG00000073125	Xlr3b	NA	-2.7	1.3	-6.1	5.07E-05	0.018361089	MAPTP301S X Tyrobp -/- vs WT
ENSMUSG00000021838	Samd4	SAMD4A	-0.6	4.6	-6.0	5.40E-05	0.019196031	MAPTP301S X Tyrobp -/- vs WT
ENSMUSG00000091089	A930018M24Rik	NA	1.6	1.3	6.0	5.57E-05	0.019431183	MAPTP301S X Tyrobp -/- vs WT
ENSMUSG00000098891	Gm27753	NA	0.9	2.0	6.0	5.82E-05	0.019930083	MAPTP301S X Tyrobp -/- vs WT
ENSMUSG00000022018	Rgcc	RGCC	0.7	3.7	5.9	6.77E-05	0.02276616	MAPTP301S X Tyrobp -/- vs WT
ENSMUSG00000083623	Gm7224	NA	1.4	1.8	5.9	6.97E-05	0.022849886	MAPTP301S X Tyrobp -/- vs WT
ENSMUSG00000041596	Nlrp5-ps	NA	-1.2	4.9	-5.8	7.13E-05	0.022849886	MAPTP301S X Tyrobp -/- vs WT
ENSMUSG00000022899	Slc15a2	SLC15A2	0.7	4.9	5.8	7.16E-05	0.022849886	MAPTP301S X Tyrobp -/- vs WT
ENSMUSG00000055341	Zfp457	ZNF93	1.3	0.3	5.8	7.52E-05	0.023615677	MAPTP301S X Tyrobp -/- vs WT
ENSMUSG00000058147	Xlr3c	NA	-3.6	-1.2	-5.8	8.10E-05	0.025022955	MAPTP301S X Tyrobp -/- vs WT
ENSMUSG00000053985	Zfp14	ZFP14	0.7	3.7	5.7	8.75E-05	0.026240388	MAPTP301S X Tyrobp -/- vs WT
ENSMUSG00000098975	Gm27177	NA	-1.4	4.7	-5.7	8.78E-05	0.026240388	MAPTP301S X Tyrobp -/- vs WT
ENSMUSG00000093954	Gm16867	NA	-1.1	5.1	-5.7	8.96E-05	0.026368036	MAPTP301S X Tyrobp -/- vs WT
ENSMUSG00000096795	Zfp433	ZNF433	-0.7	3.5	-5.7	9.60E-05	0.027809353	MAPTP301S X Tyrobp -/- vs WT
ENSMUSG00000021867	Tmem254b	TMEM254	-1.6	3.0	-5.6	9.75E-05	0.027822622	MAPTP301S X Tyrobp -/- vs WT
ENSMUSG00000095463	Entpd4	ENTPD4	-0.9	6.0	-5.6	0.000109585	0.030803201	MAPTP301S X Tyrobp -/- vs WT
ENSMUSG00000020250	Txnrd1	TXNRD1	0.4	4.9	5.5	0.000115197	0.031904602	MAPTP301S X Tyrobp -/- vs WT
ENSMUSG00000040424	Hipk4	HIPK4	-0.7	4.4	-5.5	0.000119835	0.032707956	MAPTP301S X Tyrobp -/- vs WT
ENSMUSG00000104405	Gm31748	NA	-1.1	0.9	-5.5	0.0001328	0.034734121	MAPTP301S X Tyrobp -/- vs WT
ENSMUSG00000039068	Zzz3	ZZZ3	-0.3	6.7	-5.4	0.000134767	0.034734121	MAPTP301S X Tyrobp -/- vs WT
ENSMUSG00000099136	Gm27608	NA	0.9	2.3	5.4	0.000135546	0.034734121	MAPTP301S X Tyrobp -/- vs WT
ENSMUSG00000020019	Ntn4	NTN4	0.6	3.4	5.4	0.000136424	0.034734121	MAPTP301S X Tyrobp -/- vs WT
ENSMUSG00000072676	Tmem254a	TMEM254	-1.9	-0.8	-5.4	0.00013648	0.034734121	MAPTP301S X Tyrobp -/- vs WT
ENSMUSG00000087775	Rprl2	NA	5.1	-2.4	5.4	0.00014322	0.03596338	MAPTP301S X Tyrobp -/- vs WT
ENSMUSG00000103779	Gm36931	NA	-1.0	-0.1	-5.4	0.000149409	0.037023901	MAPTP301S X Tyrobp -/- vs WT
ENSMUSG00000029071	Dvl1	DVL1	0.9	5.9	5.3	0.000170004	0.041580442	MAPTP301S X Tyrobp -/- vs WT
ENSMUSG00000098394	Gm27999	NA	0.8	2.4	5.3	0.000180584	0.043428103	MAPTP301S X Tyrobp -/- vs WT
ENSMUSG00000063808	Gpatch1	GPATCH1	-0.4	4.2	-5.3	0.000182615	0.043428103	MAPTP301S X Tyrobp -/- vs WT
ENSMUSG00000040724	Kcna2	KCNA2	-0.4	8.2	-5.2	0.000204507	0.047549031	MAPTP301S X Tyrobp -/- vs WT
ENSMUSG00000022306	Zfpm2	ZFPM2	-0.8	2.8	-5.2	0.000211824	0.048477791	MAPTP301S X Tyrobp -/- vs WT
ENSMUSG00000028104	Polr3gl	POLR3GL	0.5	2.7	5.2	0.000213776	0.048477791	MAPTP301S X Tyrobp -/- vs WT
ENSMUSG00000004151	Etv1	ETV1	-0.5	4.8	-5.2	0.000216223	0.048477791	MAPTP301S X Tyrobp -/- vs WT
ENSMUSG00000112117	RMST_1	NA	0.6	5.2	5.1	0.000224402	0.049719511	MAPTP301S X Tyrobp -/- vs WT
ENSMUSG00000099315	Gm27175	NA	-2.6	-0.5	-5.1	0.000229713	0.04988349	MAPTP301S X Tyrobp -/- vs WT
ENSMUSG00000112847	AC158605.2	NA	0.9	0.5	5.1	0.000230439	0.04988349	MAPTP301S X Tyrobp -/- vs WT
ENSMUSG00000067017	Gm3608	NA	0.4	3.9	5.1	0.000234858	0.050262265	MAPTP301S X Tyrobp -/- vs WT
ENSMUSG00000022066	Entpd4b	NA	-0.9	5.4	-5.1	0.000246052	0.05201035	MAPTP301S X Tyrobp -/- vs WT
ENSMUSG00000098009	Gm5597	NA	-0.8	0.9	-5.1	0.000255707	0.0523721	MAPTP301S X Tyrobp -/- vs WT
ENSMUSG00000084347	Akt2-ps	NA	0.8	2.1	5.1	0.00025584	0.0523721	MAPTP301S X Tyrobp -/- vs WT
ENSMUSG00000068877	Selenbp2	SELENBP1	1.0	0.0	5.0	0.000263449	0.053268622	MAPTP301S X Tyrobp -/- vs WT
ENSMUSG00000020431	Adcy1	ADCY1	-0.5	9.1	-5.0	0.000270216	0.053268622	MAPTP301S X Tyrobp -/- vs WT
ENSMUSG00000091511	Vmn2r87	NA	-0.5	2.1	-5.0	0.000272271	0.053268622	MAPTP301S X Tyrobp -/- vs WT
ENSMUSG00000008730	Hipk1	HIPK1	0.5	6.4	5.0	0.000276292	0.053268622	MAPTP301S X Tyrobp -/- vs WT
ENSMUSG00000093571	Gm24757	NA	-0.6	1.7	-5.0	0.000278157	0.053268622	MAPTP301S X Tyrobp -/- vs WT
ENSMUSG00000103753	Gm6934	NA	-1.9	-0.3	-5.0	0.000278471	0.053268622	MAPTP301S X Tyrobp -/- vs WT
ENSMUSG00000098270	Gm27221	NA	-0.8	2.5	-5.0	0.000280019	0.053268622	MAPTP301S X Tyrobp -/- vs WT
ENSMUSG00000107092	Gm7993	NA	0.8	1.7	4.9	0.000311627	0.058688772	MAPTP301S X Tyrobp -/- vs WT
ENSMUSG00000085666	Gm9855	NA	4.1	0.0	4.8	0.000376714	0.069555349	MAPTP301S X Tyrobp -/- vs WT

ENSMUSG00000098662	Gm27582	NA	0.7	2.3	4.8	0.000407123	0.074279737	MAPTP301S X Tyrobp -/- vs WT
ENSMUSG00000035594	Chrna5	CHRNA5	-0.9	1.5	-4.8	0.000410189	0.074279737	MAPTP301S X Tyrobp -/- vs WT
ENSMUSG00000023927	Satb1	SATB1	-0.4	5.3	-4.8	0.000419294	0.075205426	MAPTP301S X Tyrobp -/- vs WT
ENSMUSG00000098255	Gm27223	NA	-1.0	0.2	-4.8	0.000426974	0.075674267	MAPTP301S X Tyrobp -/- vs WT
ENSMUSG00000105843	Gm19439	NA	-1.2	1.6	-4.8	0.000431989	0.075674267	MAPTP301S X Tyrobp -/- vs WT
ENSMUSG00000076128	Mir686	NA	-0.6	4.2	-4.8	0.000434971	0.075674267	MAPTP301S X Tyrobp -/- vs WT
ENSMUSG00000021476	Habp4	HABP4	-0.3	5.6	-4.7	0.000437981	0.075674267	MAPTP301S X Tyrobp -/- vs WT
ENSMUSG00000074060	Fbxw15	FBXW12	-1.2	0.2	-4.7	0.000449559	0.076768581	MAPTP301S X Tyrobp -/- vs WT
ENSMUSG00000022325	Pop1	POP1	-0.4	3.1	-4.7	0.000452467	0.076768581	MAPTP301S X Tyrobp -/- vs WT
ENSMUSG00000097431	Gm26782	NA	-2.4	1.7	-4.7	0.00046128	0.077565126	MAPTP301S X Tyrobp -/- vs WT
ENSMUSG00000069755	Zfp125	NA	-0.9	2.7	-4.7	0.0004855	0.080915217	MAPTP301S X Tyrobp -/- vs WT
ENSMUSG00000102611	Gm37847	NA	-1.9	0.0	-4.7	0.000515984	0.084520558	MAPTP301S X Tyrobp -/- vs WT
ENSMUSG00000022193	Psmb5	PSMB5	-0.4	5.9	-4.7	0.000516108	0.084520558	MAPTP301S X Tyrobp -/- vs WT
ENSMUSG00000094786	Gm14403	NA	-0.4	3.5	-4.6	0.000526148	0.085421968	MAPTP301S X Tyrobp -/- vs WT
ENSMUSG00000026663	Atf6	ATF6	-0.4	6.3	-4.6	0.00054494	0.087716674	MAPTP301S X Tyrobp -/- vs WT
ENSMUSG00000043557	Mdga1	MDGA1	0.7	1.6	4.6	0.000560008	0.089077801	MAPTP301S X Tyrobp -/- vs WT
ENSMUSG00000024998	Plce1	PLCE1	0.4	3.9	4.6	0.000563041	0.089077801	MAPTP301S X Tyrobp -/- vs WT
ENSMUSG00000048502	Duxbl1	NA	-1.7	-0.7	-4.6	0.000567585	0.089077801	MAPTP301S X Tyrobp -/- vs WT
ENSMUSG00000098488	Pla2g4b	PLA2G4B	-0.4	3.2	-4.6	0.000574863	0.089474342	MAPTP301S X Tyrobp -/- vs WT
ENSMUSG00000019102	Aldh3a1	ALDH3A1	-0.9	-0.4	-4.6	0.000597212	0.092190927	MAPTP301S X Tyrobp -/- vs WT
ENSMUSG00000093908	Gm5784	NA	-1.1	0.3	-4.6	0.000615132	0.093425686	MAPTP301S X Tyrobp -/- vs WT
ENSMUSG00000037572	Wdhd1	WDHD1	-0.5	3.1	-4.5	0.000640014	0.096427075	MAPTP301S X Tyrobp -/- vs WT
ENSMUSG00000053898	Ech1	ECH1	-0.4	5.1	-4.5	0.000646428	0.096427126	MAPTP301S X Tyrobp -/- vs WT
ENSMUSG00000044042	Fmn1	FMN1	-0.3	6.1	-4.5	0.000651247	0.096427126	MAPTP301S X Tyrobp -/- vs WT
ENSMUSG00000063428	Ddo	DDO	-0.4	2.9	-4.5	0.000655375	0.096427126	MAPTP301S X Tyrobp -/- vs WT
ENSMUSG00000096257	Ccer2	CCER2	1.3	-0.3	4.5	0.000680344	0.099185491	MAPTP301S X Tyrobp -/- vs WT
ENSMUSG00000097509	B230322F03Rik	NA	-0.7	2.2	-4.5	0.000684655	0.099185491	MAPTP301S X Tyrobp -/- vs WT
ENSMUSG00000036915	Kirrel2	KIRREL2	0.5	2.9	4.5	0.000691804	0.099456002	MAPTP301S X Tyrobp -/- vs WT
